# A study of common Mendelian disease carriers across ageing British cohorts: meta-analyses reveal heterozygosity for alpha 1-antitrypsin deficiency increases respiratory capacity and height

**DOI:** 10.1136/jmedgenet-2015-103342

**Published:** 2016-02-01

**Authors:** Teri-Louise North, Yoav Ben-Shlomo, Cyrus Cooper, Ian J Deary, John Gallacher, Mika Kivimaki, Meena Kumari, Richard M Martin, Alison Pattie, Avan Aihie Sayer, John M Starr, Andrew Wong, Diana Kuh, Santiago Rodriguez, Ian N M Day

**Affiliations:** 1 School of Social and Community Medicine, University of Bristol, Bristol, UK; 2 MRC Lifecourse Epidemiology Unit, University of Southampton, Southampton, UK; 3 National Institute for Health Research Nutrition Biomedical Research Centre, University of Southampton and University Hospital Southampton NHS Foundation Trust, Southampton, UK; 4 National Institute for Health Research Musculoskeletal Biomedical Research Unit, University of Oxford, Oxford, UK; 5 Department of Psychology, University of Edinburgh, Edinburgh, UK; 6 Centre for Cognitive Ageing and Cognitive Epidemiology, University of Edinburgh, Edinburgh, UK; 7 Department of Psychiatry, University of Oxford, Oxford, UK; 8 Department of Epidemiology and Public Health, UCL, London, UK; 9 ISER, University of Essex, Essex, UK; 10 University of Bristol/University Hospitals Bristol NHS Foundation Trust National Institute for Health Research Bristol Nutrition Biomedical Research Unit, University of Bristol, Bristol, UK; 11 MRC Unit for Lifelong Health and Ageing at UCL, London, UK

**Keywords:** Alpha 1-Antitrypsin, Mendelian carriers, height, ALSPAC, HALCion

## Abstract

**Background:**

Several recessive Mendelian disorders are common in Europeans, including cystic fibrosis (*CFTR*), medium-chain-acyl-Co-A-dehydrogenase deficiency (*ACADM*), phenylketonuria (*PAH*) and alpha 1-antitrypsin deficiency (*SERPINA1*).

**Methods:**

In a multicohort study of >19 000 older individuals, we investigated the relevant phenotypes in heterozygotes for these genes: lung function (forced expiratory volume in 1 second (FEV1), forced vital capacity (FVC)) for *CFTR* and *SERPINA1*; cognitive measures for *ACADM* and *PAH*; and physical capability for *ACADM*, *PAH* and *SERPINA1*.

**Results:**

Findings were mostly negative but lung function in *SERPINA1* (protease inhibitor (PI) Z allele, rs28929474) showed enhanced FEV1 and FVC (0.13 z-score increase in FEV1 (p=1.7×10^−5^) and 0.16 z-score increase in FVC (p=5.2×10^−8^)) in PI-MZ individuals. Height adjustment (a known, strong correlate of FEV1 and FVC) revealed strong positive height associations of the Z allele (1.50 cm increase in height (p=3.6×10^−10^)).

**Conclusions:**

The PI-MZ rare (2%) SNP effect is nearly four times greater than the ‘top’ common height SNP in *HMGA2*. However, height only partially attenuates the *SERPINA1-*FEV1 or FVC association (around 50%) and vice versa. Height SNP variants have recently been shown to be positively selected collectively in North versus South Europeans, while the Z allele high frequency is localised to North Europe. Although PI-ZZ is clinically disadvantageous to lung function, PI-MZ increases both height and respiratory function; potentially a balanced polymorphism. Partial blockade of PI could conceivably form part of a future poly-therapeutic approach in very short children. The notion that elastase inhibition should benefit patients with chronic obstructive pulmonary disease may also merit re-evaluation. PI is already a therapeutic target: our findings invite a reconsideration of the optimum level in respiratory care and novel pathway potential for development of agents for the management of growth disorders.

## Introduction

Heterozygote carriers for recessive Mendelian (monogenic) disorders such as cystic fibrosis (MIM: 219700), medium-chain-acyl-Co-A-dehydrogenase deficiency (MIM: 201450), phenylketonuria (MIM: 261600) and alpha 1-antitrypsin (AAT) deficiency (MIM: 613490) are relatively common in the UK population (1.5% (*ACADM*) to ∼10% (protease inhibitor (PI)-MS)). Unlike in homozygote carriers, no clinical features are evident in heterozygotes although biochemical phenotype may be detectable (eg, phenylalanine level after aspartame^1^ (*PAH*)). The AAT deficiency phenotype is continuous across the six genotypes of the S and Z alleles (MM wildtype, MS, MZ, SS, SZ and ZZ). However, only individuals of ZZ genotype are notable clinically; the condition results in early-onset lung emphysema with a penetrance of 60% for ZZ individuals.^2^ There is no clear association in the literature between lung disease and individuals with either MZ^3^ or SZ genotype.^4^

The prefix PI is added to the allele or genotype name. According to this, the normal (most common) allele is PI-M and the most common pathogenic allele is PI-Z. Mendelian disease alleles such as PI-Z may be prevalent in a population through new mutation and chance, with insufficient time for fitness and selection to take effect, or through balancing selection where heterozygote advantage outweighs homozygote disadvantage. A textbook example of the latter is sickle cell anaemia where resistance to malaria confers a heterozygote advantage.

Within the Healthy Ageing across the Life Course (HALCyon) collaboration^5^ ^6^ of UK observational cohorts, we tested whether heterozygote carriers for these four Mendelian diseases exhibit phenotypic differences from non-carriers in later life. In eight studies, we genotyped the deltaF508 mutation for cystic fibrosis, the K340E mutation for medium-chain-acyl-Co-A-dehydrogenase deficiency, the three most common phenylketonuria mutations in the UK (rs5030861, rs5030858 and rs75193786 (T to C mutation)) and lastly rs28929474 and rs17580 representing PI-Z and PI-S alleles, respectively, to infer AAT PI genotypes. Lung function, cognitive capability and physical capability are complex traits that have each been shown to predict mortality.^7–9^ For homozygotes or compound heterozygotes of these four Mendelian diseases, large differences in earlier life are seen for lung function (*CFTR*, *SERPINA1*) and cognitive function (*ACADM*, *PAH*). We tested heterozygotes against equivalent later life traits accordingly, with an additional analysis of physical capability (*ACADM*, *PAH* and *SERPINA1*^10^). To generate estimates using all of the individual participant data (IPD), we pooled IPD into a single data set and conducted one-step meta-analyses of the harmonised outcomes. This is superior to a conventional two-step approach (analyses performed within each cohort and study-specific estimates pooled in a meta-analysis) when the exposure is rare.^11^ ^12^

A well-known signature of recent selection in humans is the very fast increase in frequency of the favoured allele (or haplotype) in a population.^13^ Two haplotype-based tests can detect it: the extended haplotype homozygosity (EHH) test^14^ and the integrated test iHS.^13^ Rare haplotypes are also informative. It has been suggested^15^ that reduced decay of EHH of haplotypes that are both rare and extended is informative to identify signatures of natural selection. These signatures could reflect either residual levels of an older selection phenomenon that is being diluted or an active process of natural selection.^15^ We performed an EHH analysis of rs28929474 and rs17580 using genome-wide association study (GWAS) data and PI genotype status in a UK cohort, ALSPAC.^16^ We also tested selection related to common variation around *SERPINA1* from Haplotter^13^ and estimated allele age based both on allele frequency^17^ and on local recombination between the Z locus and other SNPs in the ALSPAC data.

## Materials and methods

A list of acronyms used in this article is shown in table 1.

**Table 1:** List of acronyms

Gene acronyms	
*ACADM*	Acyl-CoA dehydrogenase, C-4 to C-12 straight chain
*CFTR*	Cystic fibrosis transmembrane conductance regulator
*HMGA2*	High mobility group AT-hook 2
*PAH*	Phenylalanine hydroxylase
*SERPINA1*	Serpin peptidase inhibitor, clade A (alpha-1 antiproteinase, antitrypsin), member 1
Outcome acronyms	
FEV1	Forced expiratory volume in 1 second
FVC	Forced vital capacity
FCRT	Four choice reaction time
Disease acronyms	
PKU	Phenylketonuria
MCADD	Medium-chain-acyl-Co-A-dehydrogenase deficiency
Cohort acronyms	
BO	Boyd Orr
CaPS	Caerphilly Prospective Study
ELSA	English Longitudinal Study of Ageing
LBC1921	Lothian Birth Cohort 1921
HAS	Hertfordshire Ageing Study
HALCyon	Healthy Ageing across the Life Course
HCS	Hertfordshire Cohort Study
NSHD	MRC National Survey of Health and Development
WHII	Whitehall II Study

### HALCyon

#### Study participants

Individuals included in this analysis belonged to the HALCyon collaboration.^5^ We meta-analysed IPD from eight UK cohorts: the Boyd Orr Cohort, the Caerphilly Prospective Study (CaPS), the English Longitudinal Study of Ageing (ELSA), the Hertfordshire Ageing Study (HAS), the Hertfordshire Cohort Study, the Lothian Birth Cohort 1921 (LBC1921), the MRC National Survey of Health and Development (NSHD) and the Whitehall II Study (WHII). Further information about the HALCyon cohorts can be found in earlier publications.^18^

#### Mutation selection

We selected the most common causal mutation to genotype for medium-chain acyl Co-A dehydrogenase deficiency (rs77931234, otherwise known as K304E or c.985A>G^19^) and cystic fibrosis (the deltaF508 mutation, rs113993960).

With the exception of the NSHD cohort, we inferred AAT PI status using the genotypes from rs28929474 and rs17580. PI-MM corresponds to an individual who is wildtype for both rs28929474 and rs17580. PI-MS individuals are wildtype for rs28929474 and heterozygous for rs17580, while PI-MZ individuals are the converse. PI-SS individuals are homozygous for rs17580 and wildtype for rs28929474, while PI-SZ individuals are heterozygous for both SNPs. PI-ZZ individuals are wildtype for rs17580 and homozygous for rs28929474. Due to their rarity, age and very close recombination distance, other genotypic combinations of rs28929474 and rs17580 would be vanishingly rare. In the NSHD, we analysed PI status measured from isoelectric focusing.^20^

Mutation selection was more complex for phenylketonuria because several hundred causal mutations have been identified to date. We selected rs5030861 (IVS12+1 G>A), rs5030858 (R408W) and rs75193786 [T to C mutation] (I65T) after consulting a review of PKU mutations in Europe^21^ and the PAH database^22^ (http://www.pahdb.mcgill.ca) and considering mutations with highest frequency in UK populations.

#### Genotyping

Genotyping was performed by LGC Genomics (http://www.lgcgenomics.com/), with the exception of rs17580 and rs28929474 in ELSA and WHII for which genotype data were already available. We inferred rs17580 and rs28929474 genotypes in the NSHD using PI classes from isoelectric focusing.^20^ Further information on the genotyping quality is provided in online supplementary table S1.

#### Harmonisation of outcomes and exposures by cohort

Wave of outcome assessment is detailed in online supplementary appendix S2. All core continuous outcomes (lung function, cognitive capability and physical capability) were transformed to z-scores by subtracting the mean and dividing by the SD of the measure within cohorts using all data available. All outcomes were further harmonised across cohorts before z-scoring, as detailed in online supplementary appendix S3.

Chronic obstructive pulmonary disease (COPD) status was determined using the Global Lungs Initiative ERS Task Force 2012 regression equations, which derive the lower limit of normal (LLN, 5th centile) values for forced expiratory volume in 1 second (FEV1) and FEV1/forced vital capacity (FVC) ratio given an individual's age, sex and height.^23^ These specify that age should be to at least one decimal place. This was not possible in ELSA, and thus, this may have introduced some error into the prediction equation. In addition, COPD status is derived in this analysis based on absolute FEV1 and FVC values rather than standardised values. Recent studies^24^ have confirmed that different apparatus are likely to result in systematic differences in lung function readings, which our categorisation of cases and non-cases for COPD has not taken into account. An individual was classed as having COPD if their FEV1/FVC ratio and their FEV1 were below the sex, height and age-specific LLN. This identified approximately 8% of individuals as having COPD, which indicated false positives as we would expect 5%.

Carrier status was defined as a binary variable in all analyses and was coded as [0] non-carrier and [1] carrier. The three *PAH* mutations were combined so that a non-carrier was homozygous for all three SNPs and a carrier was heterozygous for at least one SNP. In the analysis of PI status, separate analyses were conducted for PI-MS, PI-MZ, PI-SS, PI-SZ and PI-ZZ versus PI-MM (with PI-MM coded as 0).

Several of the outcomes were transformed prior to z-scoring to improve the normality of the residual distributions. Four choice reaction time in CaPS was inverse transformed, search speed was natural log transformed (NSHD and ELSA) and Mill Hill was squared in WHII.

Analyses of FVC were repeated with a square-root transformation and of FEV1/FVC ratio with a cube transformation. Analyses of weight and body mass index (BMI) were repeated with a natural log transformation, although these anthropometric outcomes were not z-scored.

Prior to analysis, individuals of non-European ancestry (self-reported or detected from genome-wide data) and related individuals were removed from the data set.

#### Statistical analyses

All analyses were conducted using Stata v.13.1^25^ and basic covariates were age in years and sex. Analyses considering additional covariates or conducted within strata were restricted to individuals with these covariates/information available.

The analysis of lung function by AAT PI status tested for a linear association between binary PI status (PI-MS, MZ, SS, SZ, ZZ vs PI-MM) and (1) FEV1, (2) FVC and (3) FEV1/FVC ratio. Analyses were repeated in current, ex and never smokers and in individuals classified as having COPD. Associations in all individuals were repeated with adjustment for (1) height and height-squared and (2) height, height-squared and height-cubed. Associations in COPD cases were also repeated with simultaneous adjustment for height, height-squared and smoking status. The analysis of physical capability by AAT PI status tested for association of binary PI-status with continuous or binary outcome, adjusted for age and sex.

To explore the change in effect of PI status on lung function following height adjustment, we tested for association of PI status with height (cm), weight (kg) and BMI (kg/m^2^). Associations with height were repeated with simultaneous adjustment for FEV1 and FVC.

The analysis of lung function for *CFTR* tested for an association of deltaF508 carrier status with FEV1, FVC and FEV1/FVC ratio in all individuals adjusted for age and sex, and stratified by smoking status. We also repeated the analysis in individuals classified as cases for COPD. The analysis in all individuals was repeated with simultaneous adjustment for height and height-squared.

We also tested for association of PI status (in the usual approach of PI-MS, MZ, SS, SZ, ZZ vs PI-MM) or deltaF508 carrier status with COPD case status.

The analysis of physical and cognitive capability outcomes for *PAH* and *ACADM* tested for an association of mutation carrier status with continuous or binary outcome, adjusted for age and sex.

#### Within-cohort analyses

To produce estimates by cohort, linear regression was implemented for continuous outcomes and logistic regression for binary outcomes.

#### One-step meta-analysis

A one-step meta-analysis approach using the IPD from all eight cohorts was used to derive estimates of effect sizes across all studies. This approach was adopted rather than the two-step method because the mutations are rare and thus the exposure of interest (carrier status) was often a rare event in the cohorts. One-step meta-analyses are based on the exact likelihood for the data, do not assume a normal distribution of effect estimates and do not assume that the SE of the effect estimate is exact; they are thus more appropriate in this instance.^11^ ^12^ A fixed effects (FE) or a random effects (RE) meta-analysis can be implemented within the one-step framework. We first implemented an RE meta-analysis (as described below) in all associations due to the heterogeneity in study characteristics (age, sex, geographical location). An RE model assumes that the true effect of interest differs across the populations from which the studies are sampled and estimates the average effect.

To implement a one-step RE meta-analysis for continuous outcomes, we used the following command in Stata

- mixed outcome binary_genetic_exposure i.study study#c.age study#sex || study: binary_genetic_exposure, noconstant residuals(independent, by(study)).

This mixed model tests for an RE of carrier status by cohort. The fixed portion of the model includes adjustment within cohorts for age and sex, and an intercept by cohort. Residuals are modelled to have study-specific distributions. A random intercept is not assumed.

To implement a one-step RE meta-analysis for binary outcomes, we used the following command in Stata

- *meqrlogit outcome binary_genetic_exposure i.study study#c.age study#sex || study: binary_genetic_exposure, noconstant*.

This similarly tests for a random carrier effect by cohort, with covariate adjustment within cohorts in the fixed part of the model.

The corresponding mathematical model for the continuous outcomes, with β coefficients for FEs and u coefficients for REs, as per the nomenclature in the Stata Reference Manual^26^ for mixed is

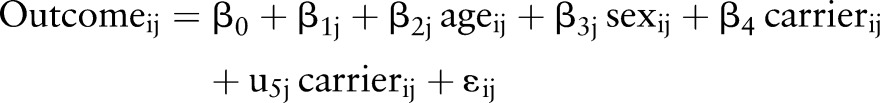

where ε_ij_ is the normally distributed residual term with mean 0 and cohort specific variance and u_5j_ is the random carrier effect by cohort with mean 0 and variance estimated by the model. The corresponding mathematical model^26^ for the binary outcomes is

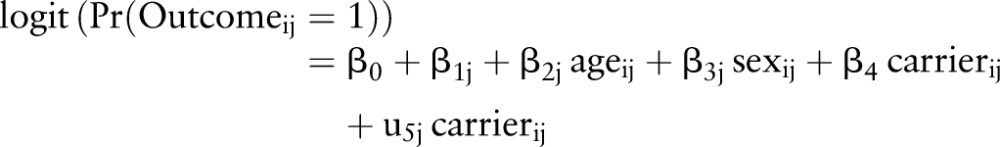


In practice, we generally found that the estimated variance of the random component of the carrier status effect (the additional effect by cohort) was negligibly small. An FE model was, therefore, more appropriate. The results presented in the main tables also include an FE model using linear regression for continuous outcomes and logistic regression for binary outcomes, pooling all of the data across cohorts, and including a dummy variable for study. In all FE models, the covariates were again included as factor variables to adjust for effects by cohort (as would be the approach in a standard two-step meta-analysis). For completeness, all tables provide the RE and the FE estimates in addition to the estimated variance of the random carrier effect for interpretation. While the variance of the RE is informative as to whether the genotypic effect was the same across cohorts, it should also be noted that the RE model for continuous outcomes assumed heteroscedastic residuals (by cohort) while the FE model used a simplification of homoscedastic residuals. In a two-step framework, heteroscedastic residuals are modelled because associations are implemented within studies before meta-analysis of the effect estimates. Our main results were robust to either implementation. For the binary outcomes of COPD status and ability to balance, we make the simplifying assumption of independent and identically distributed residuals across cohorts.

The within-cohort estimates are provided for completeness, but these often analyse a rather small number of heterozygotes (or PI-MS, MZ, SS, SZ, ZZ). The meta-analysed estimates are the most reliable as these pool the data to maximise the sample size of the carriers. Online supplementary table S2, which details sample size for the meta-analyses by outcome, should be taken into account when interpreting the coefficients.

### Selection analysis

#### Genotyping

In total, 9912 ALSPAC children were genotyped using the Illumina HumanHap550 quad genome-wide SNP genotyping platform by Sample Logistics and Genotyping Facilities at the Wellcome Trust Sanger Institute and LabCorp supported by 23andMe. Complete data for linkage disequilibrium (LD) analysis were available for 7583 unrelated individuals.

#### Statistical analyses

EHH was analysed as previously described.^14^ EHH measures the decay of homozygosity at a core haplotype of interest. Phased haplotypes involving rs28929474 and rs17580 plus 120 other SNPs (spanning ∼100 kb either side from rs28929474 and rs17580) were obtained by the software fastPHASE v1.2^27^ from 7583 ALSPAC individuals. We used the Sweep program for the identification of core haplotypes involving the two SNPs using the block definition from Gabriel *et al*.^28^

We used the Haplotter program^13^ to explore signatures of selection in the *SERPINA1* gene and surrounding genomic region (of 1 Mb either side). To this end, Haplotter considers data available for ∼800 000 common SNPs and 309 unrelated individuals from three populations. This web tool displays the results of selection from HapMap data by computing iHS, Fay and Wu's H, Tajima's D and F_st._^13^

## Results

### Meta-analysis of HALCyon cohorts

For completeness, we show both RE and FE analyses. All analyses are given as online supporting information in the order: *SERPINA1* (see online supplementary tables S3–S35), *CFTR* (see online supplementary tables S36–S40), *ACADM* (see online supplementary tables S41–S43) and *PAH* (see online supplementary tables S44–S46).

**Figure 1:**
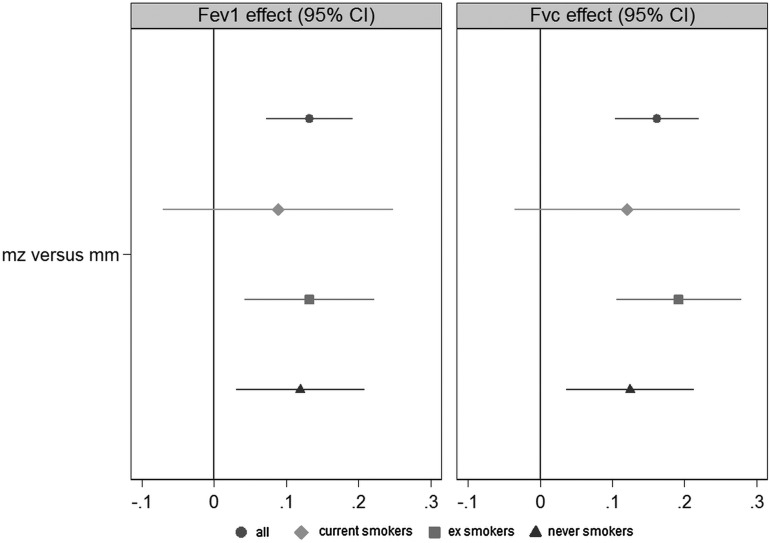
Regression coefficients from fixed effects one-step meta-analysis of protease inhibitor-MZ effect on lung function (z-scored within cohorts) adjusted for age and sex. Estimates from analyses stratified by smoking status are also provided (current smokers N=2430; ex smokers N=6422; never smokers N=5473).^38^ FEV1, forced expiratory volume in 1 second; FVC, forced vital capacity.

The genotype frequencies are provided in online supplementary tables S3–S5, S36, S41 and S44. There were no mutant homozygote calls for *CFTR*, *ACADM* or *PAH*. There was limited evidence for any carrier effect of K304E or the three *PAH* mutations combined. There was weak evidence for a negative effect of deltaF508 heterozygosity on height-adjusted FVC (see online supplementary table S37). The individual cohort and meta-analysed effect estimates for *CFTR*, *ACADM* and *PAH* are provided in online supplementary tables S37–S46. Overall for *SERPINA1*, there was no compelling evidence of an association between PI status and physical capability (see online supplementary table S24). However, there was consistent evidence across the cohorts for a respiratory difference of PI-MZ individuals compared with MM individuals (table 2). No effect was observed in PI-MS individuals. The estimated variance of the RE of carrier status on FEV1 and FVC in the RE one-step meta-analysis was very small, suggesting a fixed carrier effect across cohorts. The FEs estimate was a 0.13 SD increase in FEV1 (p=1.7×10^−5^) and a 0.16 SD increase in FVC (p=5.2×10^−8^) using IPD data from all eight cohorts. Taking the study SDs and multiplying by these coefficients, this corresponds to a difference of approximately 81–108 mL (FEV1) and 115–170 mL (FVC). There was no association with FEV1/FVC ratio (see online supplementary table S6). Our analysis of the possible effect of smoking is shown in figure 1 (see online supplementary tables S10–S15). Stratifying as current (N=2430), ex (N=6422) and never (N=5473) smokers, there was no evidence for a difference in PI-MZ effect by smoking status.

**Table 2:** Association of alpha 1-antitrypsin protease inhibitor (PI) status with standardised lung function adjusted for age and sex

		Regression coefficient (95% CI)	
Outcome	Cohort	MS vs MM	MZ vs MM†
Maximum FEV1	BO	−0.05 (−0.36 to 0.27)	0.45 (−0.14 to 1.04)
	CaPS	0.07 (−0.10 to 0.24)	0.11 (−0.12 to 0.33)
	ELSA	0.05 (−0.03 to 0.12)	0.12* (0.01 to 0.23)
	HAS	−0.49* (−0.94 to −0.05)	0.08 (−0.39 to 0.55)
	HCS	−0.01 (−0.10 to 0.09)	0.05 (−0.10 to 0.19)
	LBC1921	−0.06 (−0.31 to 0.19)	0.16 (−0.22 to 0.54)
	NSHD	0.02 (−0.09 to 0.13)	0.18* (0.01 to 0.35)
	WHII	0.05 (−0.03 to 0.12)	0.18** (0.06 to 0.29)
	Combined FE	0.03 (−0.01 to 0.07)	0.13**** (0.07 to 0.19)
	Combined RE	0.03 (−0.01 to 0.07)	0.13**** (0.07 to 0.19)
	Estimated var‡	1.20e−13 (0.00e+00 to .)	1.09e−19 (0.00e+00 to .)
Maximum FVC	BO	0.03 (−0.26 to 0.32)	0.43 (−0.11 to 0.97)
	CaPS	0.08 (−0.10 to 0.26)	0.15 (−0.09 to 0.39)
	ELSA	0.02 (−0.05 to 0.09)	0.12* (0.01 to 0.22)
	HAS	−0.35 (−0.73 to 0.03)	0.33 (−0.09 to 0.74)
	HCS	−0.02 (−0.11 to 0.07)	0.14* (0.01 to 0.27)
	LBC1921	−0.07 (−0.32 to 0.17)	0.20 (−0.17 to 0.56)
	NSHD	0.02 (−0.09 to 0.13)	0.20* (0.04 to 0.37)
	WHII	−0.01 (−0.08 to 0.07)	0.19** (0.07 to 0.30)
	Combined FE	0.01 (−0.03 to 0.04)	0.16**** (0.10 to 0.22)
	Combined RE	0.00 (−0.04 to 0.04)	0.16**** (0.10 to 0.22)
	Estimated var‡	3.76e−15 (1.01e−28 to 1.40e−01)	3.53e−19 (0.00e+00 to .)

Estimates for PI-SS, SZ and ZZ are provided in the online supplement.

*p<0.05, **p<0.01, ***p<0.001, ****p<0.0001.

†Exact p values for PI-MZ-FEV1 were 1.7×10^−5^ (FE) and 1.1×10^−5^ (RE); for PI-MZ-FVC were 5.2×10^−8^ (FE) and 3.2×10^−8^ (RE).

‡Estimated variance of the random slope on carrier status modelled by the RE model.

BO, Boyd Orr; CaPS, Caerphilly Prospective Study; ELSA, English Longitudinal Study of Ageing; FE, fixed effect; FEV1, forced expiratory volume in 1 second; FVC, forced vital capacity; HAS; Hertfordshire Ageing Study; HCS, Hertfordshire Cohort Study; LBC1921, Lothian Birth Cohort 1921; NSHD, MRC National Survey of Health and Development; RE, random effect; WHII, Whitehall II Study.

Considering the well-known correlation of lung function with height,^23^ additional models adjusted for height were run for the AAT variants (see online supplementary table S17). We initially adjusted for height and height-squared (theoretically considering respiratory surface area), with additional adjustments for height-cubed (theoretically considering total respiring cell mass; see online supplementary table S22). Empirically, FEV1 and FVC depend on powers of height in the range 2.1–2.4 (Global Lung Function Initiative prediction equations^23^). The association of PI-MZ status with FEV1 and FVC was attenuated after adjustment for powers of height (height and height-squared, table 3), but approximately half of the effect remained, suggestive that height and lung function are partially related covariates of PI-MZ. Including height-cubed did not further attenuate the genotypic association. We also considered the unadjusted PI-MZ association (FE meta-analysis) with percentage of predicted FEV1 or FVC using the Global Lungs Initiative ERS Task Force 2012 regression equations^23^ used in the COPD classification. This resulted in a slight attenuation of the association with FEV1 (1.3% increase, p=0.09) and FVC (1.6% increase, p=0.02). While the prediction equations could be accounting for height in a purer way to covariate adjustment, they produced percentage of predicted values lower than 100% in HALCyon never smokers, which indicates that prediction equations specific to this sample of British ageing individuals of European ancestry may be required. The question of whether PI-MZ exerts a pleiotropic effect of enhanced respiratory capacity independently of its height association thus requires further investigation.

**Table 3:** Association of alpha 1-antitrypsin protease inhibitor (PI)-MZ status and standardised lung function adjusted for age, sex, height and height-squared

Outcome	Cohort	Regression coefficient (95% CI)
Maximum FEV1	BO	0.17 (−0.40 to 0.74)
	CaPS	0.09 (−0.12 to 0.30)
	ELSA	0.06 (−0.05 to 0.16)
	HAS	0.11 (−0.34 to 0.56)
	HCS	−0.01 (−0.15 to 0.12)
	LBC1921	0.09 (−0.27 to 0.44)
	NSHD	0.08 (−0.08 to 0.23)
	WHII	0.11 (−0.00 to 0.21)
	Combined FE	0.07* (0.01 to 0.12)
	Combined RE	0.07* (0.01 to 0.12)
	Estimated var†	1.03e−17 (1.80e−32 to 5.87e−03)
Maximum FVC	BO	0.06 (−0.43 to 0.56)
	CaPS	0.13 (−0.08 to 0.34)
	ELSA	0.04 (−0.06 to 0.14)
	HAS	0.35 (−0.04 to 0.74)
	HCS	0.07 (−0.05 to 0.18)
	LBC1921	0.10 (−0.23 to 0.44)
	NSHD	0.08 (−0.06 to 0.23)
	WHII	0.10 (−0.00 to 0.20)
	Combined FE	0.08** (0.03 to 0.13)
	Combined RE	0.08** (0.03 to 0.13)
	Estimated var†	1.25e−13 (5.20e−27 to 3.01e+00)

*p<0.05, **p<0.01, ***p<0.001, ****p<0.0001.

†Estimated variance of the random slope on carrier status modelled by the RE model.

BO, Boyd Orr; CaPS, Caerphilly Prospective Study; ELSA, English Longitudinal Study of Ageing; FE, fixed effect; FEV1, forced expiratory volume in 1 second; FVC, forced vital capacity; HAS; Hertfordshire Ageing Study; HCS, Hertfordshire Cohort Study; LBC1921, Lothian Birth Cohort 1921; NSHD, MRC National Survey of Health and Development; RE, random effect; WHII, Whitehall II Study.

**Figure 2:**
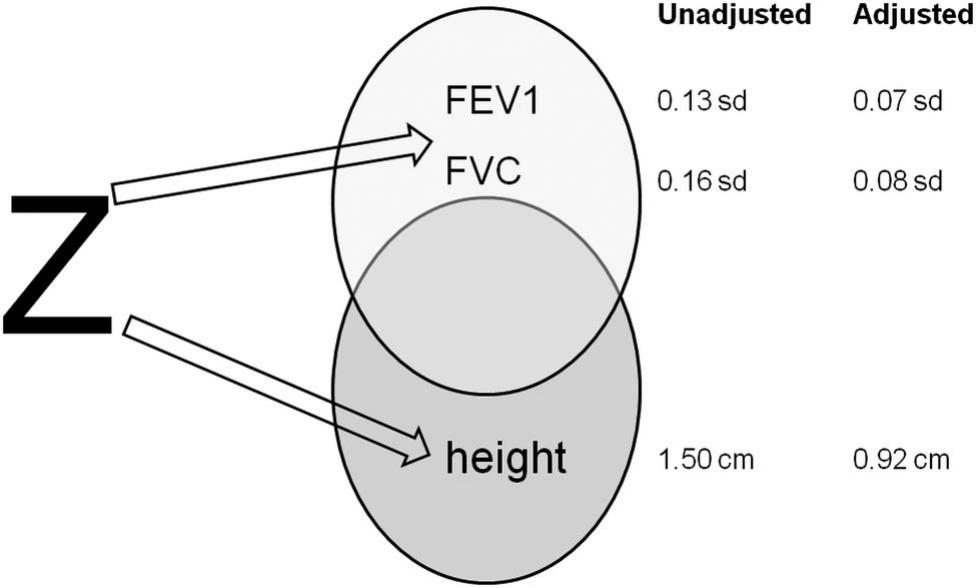
The pleiotropic effect of the Z allele on respiratory capacity and height. ‘Unadjusted’ estimates are from the (upper circle) one-step fixed effects analyses of z-scored forced expiratory volume in 1 second (FEV1) or z-scored forced vital capacity (FVC) on protease inhibitor (PI)-MZ versus PI-MM adjusted for age and sex or (lower circle) from the one-step fixed effects analysis of height (cm) on PI-MZ versus PI-MM adjusted for age and sex. The ‘adjusted’ estimates are additionally adjusted for (upper circle) height (cm) and (lower circle) z-scored FEV1 and z-scored FVC simultaneously.

The linear association between PI-MZ and height, adjusted for age and sex (table 4), was notable (p=3.6×10^−10^, FE analysis) but was not observed for PI-MS. MZ individuals averaged approximately 1.5 cm taller than MM individuals. The FE and RE meta-analyses were repeated in individuals <55 years of age. The coefficient was reduced slightly (1.3 cm increase in MZ, p=0.005, n=4552 FE analysis of four cohorts), but contained the CI including all eight cohorts. We, therefore, concluded that the PI-MZ effect on height represents a growth not age-related shrinkage effect. There is also some hint (see online supplementary table S27) that mean height may increase across genotypes MM;MZ;SZ;ZZ. The association of PI-MZ versus MM and height was additionally simultaneously adjusted for FVC and FEV, which attenuated but did not remove the association (see online supplementary table S29). We note that both the respiratory and height associations occur in geographically confined cohorts. The by-cohort analyses show no evidence for a geographically stratified effect. The PI-MZ age-adjusted and sex-adjusted associations with height, FEV1 z-score and FVC z-score did not appear to be driven by population stratification when we ran models adjusted for principal components in four of the studies (subsamples of ELSA, WHII, CaPS and LBC1921 with principal components available), although sample size was markedly attenuated. A homogeneity analysis (χ^2^ contingency test) to test whether genotype frequencies of AAT deficiency PI status differ among cohorts did not reveal significant heterogeneity (p=0.310). Nominal but minor differences between observed and expected genotype frequencies were observed for HAS (contributions to χ^2^>3.84), but these are related to low numbers and may be explained as type I error. We concluded that the PI-Z allele may have pleiotropic effects on height and respiratory function (figure 2).

**Table 4:** Association of alpha 1-antitrypsin protease inhibitor (PI) status and height (cm) adjusted for age and sex

	Regression coefficient (95% CI)	
Cohort	MS vs MM	MZ vs MM†
BO	2.31 (−0.24 to 4.86)	7.33** (2.36 to 12.30)
CaPS	0.27 (−0.97 to 1.51)	0.55 (−1.08 to 2.18)
ELSA	0.20 (−0.40 to 0.81)	2.02**** (1.12 to 2.92)
HAS	0.24 (−3.04 to 3.51)	−0.26 (−3.69 to 3.18)
HCS	−0.10 (−0.87 to 0.67)	1.23* (0.09 to 2.37)
LBC1921	0.44 (−1.55 to 2.43)	1.06 (−1.89 to 4.00)
NSHD	0.68 (−0.11 to 1.47)	1.84** (0.64 to 3.04)
WHII	0.23 (−0.34 to 0.81)	1.24** (0.34 to 2.14)
Combined FE	0.28 (−0.03 to 0.59)	1.50**** (1.03 to 1.97)
Combined RE	0.28 (−0.03 to 0.59)	1.51**** (1.04 to 1.97)
Estimated var‡	2.77e−13 (1.06e−26 to 7.27e+00)	1.67e−12 (1.59e−25 to 1.76e+01)

Estimates for PI-SS, SZ and ZZ are provided in the online supplement.

*p<0.05, **p<0.01, ***p<0.001, ****p<0.0001.

†Exact p values for PI-MZ—height were 3.6×10^−10^ (FE) and 2.9×10^−10^ (RE).

‡Estimated variance of the random slope on carrier status modelled by the RE model.

BO, Boyd Orr; CaPS, Caerphilly Prospective Study; ELSA, English Longitudinal Study of Ageing; FE, fixed effect; HAS; Hertfordshire Ageing Study; HCS, Hertfordshire Cohort Study; LBC1921, Lothian Birth Cohort 1921; NSHD, MRC National Survey of Health and Development; RE, random effect; WHII, Whitehall II Study.

The association of PI-MZ with weight and BMI was assessed (see online supplementary tables S31–S35). We observed no association for BMI and an effect estimate for weight that was consistent with what is predicted given the observational correlation between height and weight.

A previous population-based study showed a lower FEV1 in PI-MZ compared with PI-MM in individuals with clinically defined COPD, adjusted for age, sex, height and smoking status.^29^ Using the Global Lungs Initiative ERS Task Force 2012 regression equations,^23^ we classified all individuals as either cases or non-cases for COPD and reran the age-adjusted and sex-adjusted model in COPD cases (see online supplementary tables S16, S19 and S20). We did not replicate the results of the previous study, nor after adjustment for powers of height and smoking status. We meta-analysed the odds of COPD in PI classes compared with MM individuals (see online supplementary tables S25 and S26). There was no compelling evidence for an association with PI-MS or PI-MZ, while the PI-SS, SZ and PI-ZZ meta-analyses were not possible due to the low genotype frequencies. However, two out of six ZZs with available data displayed COPD (one extreme).

Online supplementary table S2 shows that overall we conducted in the region of 182 one-step meta-analyses across all outcomes and genetic variants. Many of these tests were not independent due to the outcomes (eg, FEV1/FVC ratio is derived from FEV1 and FVC) or the genetic exposures (eg, PI-MM were included in all PI analyses) or due to subgroup analyses (eg, smokers, COPD) or rerunning adjusted models. However, even with a Bonferroni adjustment based on this number (p=0.00027), our main results still produce comparatively small p values. The results of further sensitivity analyses are described in online supplementary appendix S4.

### Selection analysis

EHH results involving rs28929474 and rs17580 show small decay of EHH, from 1 to 0.6, after 90 kb from 5′ (see online supplementary figure S1). This relatively small reduction is observed for two rare haplotypes (of 5% and 2% frequency, respectively) each of them including the rare allele of each SNP. The decay of EHH is more pronounced to the 3′ end, with EHH for both rare haplotypes being reduced to 0.5 at a distance of 30 kb. These results were qualitatively unchanged with the addition of neighbouring SNPs to the core region.

Results observed from Haplotter for common SNPs analysed by iHS, Fay and Wu's H, Tajima's D and F_st_ show no evidence of selection driving alleles at intermediate or high frequency in and around the *SERPINA1* gene (see online supplementary figure S2).

Recombination data from the ALSPAC sample combined with pairwise LD between SNPs around rs28929474 suggested an allele age of between 100 and 250 generations (see online supplementary figure S3). In contrast, using Z allele frequency this estimate was 1758 generations.

## Discussion

The PI-MZ rare (2%) SNP height effect is about fourfold greater than that for the top common SNP in *HMGA2* for height. However, PI-MZ is not represented on GWAS chips, so the largest height meta-analyses of up to 250 000 individuals^30^ would not have detected it directly and apparently did not do so by imputation. Furthermore, whole-genome sequencing studies such as UK10K (http://www.UK10K.org) would not have analysed enough individuals to robustly detect the effect even if calls and imputation on low read depth were efficient. While analyses of the possible contribution of common SNPs to height suggest that they could explain the large part of this highly heritable trait,^31^ our observation raises the possibility that many common SNPs might be each weakly proxying rarer causal alleles.

Our main results of interest (MZ carrier effect on lung function and height) were obtained from a large number of carriers (>600) at the meta-analysis level. Neither association explains the other, although there is partial phenotypic correlation. The enhancement (rather than reduction) of FEV1 and FVC by PI-Z allele heterozygosity was unexpected and is in apparent contrast with the suggestion of greater incidence of respiratory infections in PI-MZ children^20^ and with the well-known severe deleterious effects of PI-ZZ. However, mechanisms for balancing selection on PI-MZ (rs28929474) have previously been proposed,^32^ and the potential connective tissue and immunological/inflammatory effects of the Z allele^32^ could plausibly lead to enhanced FEV1 and FVC with either positively or negatively correlated inflammatory or infection susceptibility. Previous studies have detected an interaction of PI-MZ with smoking such that PI-MZ ever smokers have reduced respiratory capacity compared with PI-MM.^33^ Our analysis restricted to current smokers did not detect reduced respiratory capacity in this group of individuals, and we observed enhanced respiratory capacity in ex smokers. Seventeen per cent of individuals with the relevant covariates (PI status, lung function, age and sex) were current smokers in HALCyon, 97 of which were PI-MZ. Future observational studies with increased sample size should consider current or ever smoking PI-MZ individuals to consider whether there is reduced respiratory capacity in this subgroup of individuals. Alternatively, it could be that a cumulative smoke exposure of an as yet undetermined amount determines the development of respiratory disease in PI-MZ individuals; there is evidence in PI-ZZ and PI-SZ individuals that such a concept exists.^4^ ^34^ Consequently, future studies may also need to quantify relevant environmental exposures such as cigarette smoking.

Microsatellite dating of the Z allele suggests appearance 107–135 generations ago, with high prevalence in North Europe.^35^ Height SNP variants have recently been shown collectively to have been positively selected in North (vs South) Europeans.^36^ Using GWAS data and PI genotype status in another UK cohort, ALSPAC,^16^ we analysed for EHH (see online supplementary figure S1). We also tested selection related to common variation around *SERPINA1* from Haplotter^13^ (see online supplementary figure S2) and estimated allele age based both on allele frequency^17^ and on local recombination between the Z locus and other SNPs. Recombination data in conjunction with pairwise LD between SNPs around rs28929474 indicate an allele age consistent with earlier microsatellite estimates (from 100 to 250 generations, see online supplementary figure S3), and even for a rare SNP, the haplotypes on which Z and S reside are extended, whereas Z allele frequency estimates an age about 10× older (1758 generations). These genomic features all point towards positive selection acting on the Z (and S) alleles. It is, therefore, possible that PI-Z, here shown to be a rarer allele for greater height, has been positively selected on height (or weight—a possible survival advantage in colder latitudes) though PI-ZZ is detrimental to respiratory health. PI-MZ may thus represent a balanced polymorphism with greater height or FEV1 or FVC being advantageous in heterozygotes but lung (and liver) disease being disadvantageous in ZZ homozygotes.

AAT is a therapeutic agent and target in relation to its respiratory importance.^37^ Our findings in PI-MZ heterozygotes invite both a reconsideration of what may be an optimal level of AAT for best respiratory function and for the first time a consideration whether AAT may mark a novel aspect of height determination, which could itself become a therapeutic target for height modification in some growth deficiency disorders.

## Supplementary Material

Web supplement
