# Supplementary material for: A study of common Mendelian disease carriers across ageing British cohorts: meta-analyses reveal heterozygosity for alpha 1-antitrypsin deficiency increases respiratory capacity and height
Source: J Med Genet. 2016 Feb 1;53(4):280–8. doi: 10.1136/jmedgenet-2015-103342 (PMC4819619; doi:10.1136/jmedgenet-2015-103342)
Supplement: Web supplement [file jmedgenet-2015-103342-s1.pdf]

S1 Supporting Information

S1 Appendix. Ethical Approval

BO: Ethical approval for the clinical third wave of follow-up of Boyd Orr (2002-03) was obtained from Multi-centre Research Ethics Committee Scotland. All participants gave informed consent.

CaPS: Ethical approval for genotypic analyses was provided by South East Wales Local Research Ethics Committee Panel B (05/WSE02/131). The original CaPS project received ethical approval from the former South Glamorgan Area Health Authority.

English Longitudinal Study of Ageing (ELSA): ELSA has been approved by the National Research Ethics Service and all participants have given informed consent.

HCS/HAS: Ethical approval for the Hertfordshire studies was obtained from the Hertfordshire Local Research Ethics Committee.

LBC1921: Ethical approval for the Lothian Birth Cohort 1921 study was given by the Lothian Research Ethics Committee.

NSHD: Ethical approval for the NSHD data collection at 53 years was approved by the North Thames Multi-Centre Research Ethics Committee (ref. MREC 98/1/121). At 60–64 years ethical approval was obtained from the Central Manchester Local Research Ethics Committee (ref. 07/H1008/245) and the Scotland A Research Ethics Committee (ref. 08/MRE00/12). Written informed consent was obtained from study members at each stage of data collection.

Whitehall II: All participants provided written consent and the University College London ethics committee approved the study.

ALSPAC: Ethical approval for the study was obtained from the ALSPAC Ethics and Law Committee and the Local Research Ethics Committees. Written informed consent for the study was obtained for genetic analysis.

S2 Appendix. Wave of outcome assessment

Boyd Orr (BO)(2): Physical capability and lung function were assessed at the third wave (2002-03).

Caerphilly Prospective Study (CaPS): Cognitive function measures were assessed at phase III. Physical capability measures were assessed at phase V. Lung function measures were analysed from phase I where available, but were substituted with measures from phase II for those individuals who did not have valid measures at phase I but did at phase II.

English Longitudinal Study of Ageing (ELSA)(3): Physical capability, cognitive capability and lung function were assessed at wave 2 (2004/5).

Hertfordshire Ageing Study (HAS) (4): Cognitive function was assessed at wave 1 (1994/5). Grip strength was assessed at wave 1 and all other physical capability measures at wave 2. Lung function was measured at wave 2 (2003/05).

Hertfordshire Cohort Study (HCS) (5): Grip strength and lung function were assessed at wave 1 (1999-2004) while TUG (Timed-Up and Go) speed, walk speed, balance ability and chair rise speed were assessed at both waves 1 (1999-2004) and 2 (2004/05) with partial overlap in some tests and no overlap in others. These latter measures were combined across waves, with priority given to wave 1, and the covariates tailored as such.

Lothian Birth Cohort 1921 (LBC1921) (6): Physical and cognitive capability and lung function were assessed at wave 1 age 79 years.

MRC National Survey of Health and Development (NSHD) (7): All cognitive capability and lung function measures were taken from the 1999 wave when the study members were 53

years. All physical capability measures were taken from the 1999 wave with the exception of TUG speed, which was analysed when the study members were 60-64 years.

Whitehall II Study (WHII) (8): Walking speed and lung function were analysed at phase 7 (2002-04), while all cognitive outcomes were analysed at phase 5 (1997-99).

S3 Appendix. The derivation and harmonization of variables

Physical capability

Details of the ascertainment and harmonisation of the five measures of physical capability used in analyses are described in detail elsewhere(9) and are summarised here. The approach to harmonise chair rise times (5 or 10 rises) was to calculate chair rise speed in the current study.

Grip strength was tested in ELSA, HAS, HCS, LBC1921 AND NSHD using handheld dynamometers (the specific devices used in each study are described elsewhere (9)). The maximum measure was used in each study (extracted from 3 measures of each hand in ELSA, HAS and HCS, 3 measures of the dominant hand in LBC1921 and 2 measures in each hand in NSHD). If repeat measures were missing the existing measures were used to derive the maximum.

Standing balance was assessed in BO, CaPS, ELSA, HAS, HCS and NSHD. Owing to the heterogeneity in the way the test was administered across cohorts, the outcome used in analyses was a derived binary variable for inability to balance on one leg with eyes open for five seconds. In ELSA the tests administered were more complex as described by Cooper *et al.*(9) and we derived the outcome in the same way, namely, inability to balance in full tandem with eyes open for 5 seconds with individuals who were not progressed to the next phase of testing classed as unable. Individuals who did not complete the balance test for health reasons were classed as unable in all analyses. If tests were conducted more than once the best performance was used to derive the outcome variable.

The timed walk test was conducted in LBC1921 (6 metres as fast as possible), HAS and HCS (3 metres at normal pace), ELSA (8 feet at normal pace with 2 trials) and WHII (8 feet at

normal pace with 3 trials). To normalise the distribution and to make a higher outcome a healthier outcome, times were converted to speeds in metres per second and then averaged where repeat trials were available.

The timed get up and go test was performed in BO, HAS, HCS, CaPS and NSHD. In all cohorts, study members had to rise from a chair, walk 3 metres at a normal pace and return to a seated position in the chair. The test was repeated in BO and CaPS. Again all times were converted to speeds in metres per second and then averaged where the trial was conducted more than once.

Timed chair rises were assessed in HAS, HCS, ELSA and NSHD. All times were converted to chair rise speed in stands per second. The cohorts measured time to complete 5 or 10 chair rises as fast as possible. In ELSA, individuals under 69 years performed 10 rises while those aged 70 and over performed 5 rises. Time to complete 5 rises was measured in both age groups and this was used to derive chair rise speed.

Some physical performance measures were conducted in part of the HCS cohort in one wave and in the remaining cohort in a later wave. To maximise sample size, measures were pooled across waves and covariates were tailored according to the wave at which the outcome had been performed.

Cognitive capability

The measures of cognition across the HALCyon cohorts were categorised into measures of crystallised ability and measures of fluid cognition.

Measures of crystallised cognitive function

The National Adult Reading Test (NART)(10) was available in LBC1921, CaPS and NSHD. This requires study members to read aloud 50 words with irregular pronunciation and the number of words pronounced correctly is used in analyses here. NART should reflect pre-morbid IQ.

The Mill Hill vocabulary test(11) was administered in HAS and WHII. Study members had to choose the correct synonym for 33 words out of 6 multiple choice answers with increasing difficulty. The number of correct answers is used in analyses.

Measures of fluid cognitive function

Semantic fluency was tested in ELSA, NSHD and WHII via a verbal or written test where study members were asked to name as many animals as possible in 1 minute. The number of unique animals named was used in analyses.

Verbal memory was tested in ELSA, NSHD and WHII via a word recall test. The numbers of words correctly recalled was used in analyses. In NSHD, we summed the total score for remembering the same 15 words in writing over three consecutive trials. The sum of two trials with a delay for the second trial for remembering 10 words verbally was analysed in ELSA. 20 words were recalled in writing in WHII.

Phonemic fluency was analysed in LBC1921 and WHII. In LBC1921, study members were given three 1 minute trials to name as many words as possible beginning with F, L and C. The total number of words is used in analyses. In WHII, study members wrote as many words as possible in 1 minute beginning with S.

Search speed was tested in ELSA (780 letters) and NSHD (600 letters) whereby participants crossed out particular letters in a large grid of letters. The number of letters searched per minute was used in analyses.

The Alice Heim 4-I test (AH4)(12) was available for analyses in CaPS, HAS and WHII. This involves 65 verbal and mathematical questions. The total score achieved in 10 minutes was used in analyses here.

Choice reaction time (FCRT) was assessed in CaPS via a computer test in which the study members had to press one of four key pads depending on which box a stimulus appeared in on screen.

Wechsler logical memory(13) was tested in LBC1921. The participants were asked to recall two stories immediately and following a delay for each. The total sum of the scores for each story were progressed to analysis.

Raven's Progressive Matrices(14) were used in LBC1921, in which study members were given 20 minutes to complete 60 multiple choice "complete the pattern" questions. The total score was used in the analysis.

Lung function

We analysed Forced Expiratory Volume in 1 second (FEV1) and Forced Vital Capacity (FVC). All measures for which an unsatisfactory technique had been recorded were removed. All measures were cleaned so that values <0.3 litres and >9 litres were removed. Any instances for which FVC was less than FEV1 were changed to missing (both values excluded from the analysis). If a cohort provided the individual repeat trial data, the cleaning was applied to each trial; if a cohort provided a cleaned summary measure across trials, this was applied to these values.

If a study provided data from individual trials, the maximum FEV1 and the maximum FVC were derived. These could come from different trials. The FEV1/FVC ratio was derived by taking the ratio of these maxima. Individuals were only included in the analysis of lung function if they had both a maximum FEV1 and a maximum FVC. The FEV1, FVC and FEV1/FVC ratio values (derived from the maxima of repeat trials) were z-scored within cohorts to have a mean of 0 and a standard deviation of 1 using all data available.

In the Boyd Orr cohort, lung function was assessed using a compact II Vitalograph Spirometer. Up to 5 blows were conducted per study member and provided for analysis.

In CaPS, the values provided were the maximum FEV1 from 3 trials and the maximum FVC from 3 trials when the highest two valid FEV1 (and separately the highest two valid FVC) were within 100ml of each other. These were derived by Bolton *et al*. and spirometry was performed in the standing position using a McDermott spirometer(15).   Values from phase 1 were preferentially used but values from phase 2 were substituted into the analysis if the phase 1 measures were missing or removed by cleaning. Covariates were tailored as such.

ELSA provided the highest technically satisfactory FEV1 reading and the highest technically satisfactory FVC reading (both in litres). These had been derived using data across 3 blows using a Vitalograph Escort spirometer.

In LBC1921, lung function was measured with 3 blows of a MicroMedical Spirometer in the sitting position without nose clips. The best FEV1 and FVC of the three blows were provided by the cohort for analysis.

In HAS, FEV1 and FVC were provided for 2 blows using a MicroMedical Micro Spirometer(4).

Lung function in HCS was measured using a MicroMedical Micro Spirometer(5). Three trials were conducted and provided for analysis.

In NSHD, lung function was measured using a MicroMedical Plus Spirometer(16), the cohort provided values as the maximum of two blows (for FEV1 and FVC separately) when the difference between trials was less than or equal to 0.30 L. If the study member only had one valid measure they were excluded from analyses. Biologically unfeasible values (<0.30L or >9L) and individuals regarded as having an unsatisfactory technique were removed before deriving the maxima.

Lung function was measured in WHII using a MicroMedical Micro Plus Spirometer. Each study member attempted three blows which were provided for analysis.

Height, weight and BMI

Standing height (cm), weight (kg) and Body Mass Index (BMI, kg/m^2) were included in analyses. In CaPS, height was taken from phase 1 and replaced with height from phase 2 if the phase 1 measure was missing. For the PI-height analyses, a tailored age variable for the wave at which height was assessed was derived. In Boyd Orr, height, weight and BMI were analysed from the clinic sample.

Age and smoking covariates

Individuals aged 90 years or over are not assigned an exact age in ELSA data releases. As such, we estimated the age of these individuals using a representative estimate of the mean age of individuals aged 90 and over in England and Wales in 2005 (the year of wave 2 assessment). To calculate this estimate, we used the England and Wales Mid-Year Population Estimates of the Very Elderly, 2002-2010, demographic table "Mid-2010 Estimates of the very elderly (including centenarians) England and Wales; estimated resident population"

which was part of the Population Estimates of the Very Elderly, 2010 Office for National Statistics release (release date 29 September 2011, date accessed 5 February 2014 from http://www.ons.gov.uk/ons/publications/re-reference-tables.html?edition=tcm%3A77-223697). The estimated age used in analyses was 92.62 years. These individuals were not included in analyses of lung function or anthropometry.

Smoking status was defined as current, ex or never smoker; pipe and cigar smoking was included if this information was available.

S4 Appendix. Sensitivity analyses

The association between PI-MZ and lung function was repeated (basic model, height adjusted model) with FEV1 and FVC values outside +/- 3 standard deviations removed within cohorts before z-scoring to explore the robustness of the coefficient estimates to extreme values, S8 and S21 Tables. This was also implemented for the PI-MZ and height associations (basic model, lung function adjusted model) and the PI-MZ and weight association (S28, S30 and S32 Tables).

We repeated the basic PI-MZ vs PI-MM random effects models against FEV1, FVC and height using restricted maximum likelihood rather than the default maximum likelihood. This made negligible difference to the fixed effects estimates and p-values, while the variance of the random carrier effect was still immaterial.

The distribution of the raw, standardised and studentised residuals were reviewed from the fixed-effects analyses to examine the normality of the distribution. The residuals versus the fitted values were examined for all models suggestive of an association to check for independence. The fixed effects linear model of PI-MZ and z-scored FEV1 suggested that the variance of the residuals increased slightly with the fitted values, but a transformation of the outcome would not remediate this. The distribution of the residuals of FEV1 tended to have a fat tail for negative residuals, for which there was no appropriate transformation.

We repeated the fixed effects analysis of PI-MZ vs MM against z-scored FEV1 and FVC (age and sex adjusted) with robust standard errors, which do not assume that the residuals in the model are identically distributed. The regression coefficients do not change with this approach but the updated 95% confidence intervals were (0.07, 0.19) for FEV1 and (0.10, 0.22) for FVC. The p-values were very slightly attenuated.

We considered whether the variance in lung function (z-scored FEV1 and FVC, square-root transformed and z-scored FVC) differed across PI classes and in deltaF508 carriers vs non-

carriers (FVC), in addition to whether the variance in height (cm) differed across PI classes by pooling the data across cohorts and using Levene's test (S47 Table). This revealed that there was evidence for a difference in the variance of FEV1 and FVC in PI-MZ vs MM (greater variance in PI-MZ). However, the difference was reasonably small (difference of 0.06 in the standard deviation of pooled z-scored FEV1 and difference of 0.08 in the standard deviation of pooled z-scored FVC).

To account for the unequal variance in PI-MZ vs PI-MM, we repeated the basic fixed effects model (age and sex adjusted) with z-scored FEV1 and FVC within a mixed effects framework, enforcing heteroskedastic residuals by genotype rather than by cohort (so the only random component of the model is the clustering of residuals by genotype). The effect estimates were unchanged but the p-values were slightly attenuated ($p=2.6 \times 10^{-5}$ (FEV1) and $p=1.3 \times 10^{-7}$ (FVC)).

We considered influential observations for the associations of interest. These were (1) PI-MZ vs MM and FEV1 (basic and height, height-squared adjusted models) (2) PI-MZ vs MM and FVC (basic and height, height-squared adjusted models) (3) PI-MZ vs MM and height (basic and lung function adjusted models) (4) PI-MZ vs MM and natural log transformed weight (5) deltaF508 carrier status (height, height-squared adjusted) and FVC and (6) PI-MZ vs MM and grip strength. We calculated the dfbeta statistics from the fixed effects meta-analyses. These calculate by how many standard errors the regression coefficient would change if a single observation were omitted. This confirmed that no observations had a dfbeta value with magnitude greater than or equal to 1, such that no single observation affected the coefficient estimate by more than one standard error. However, we did note that the usual threshold of 2/sqrt(sample size)(1) was extremely sensitive and identified >60% of the mutation carriers for each regression. Owing to the large sample size (>14,000 observations for all analyses except the grip strength analysis which included >10,000 individuals), this

identified observations which changed the coefficients by approximately 0.017-0.02 standard errors. Given the small standard errors in these associations, this identified observations which changed the regression coefficient by only a small amount. A summary of the range of dfbeta values by analysis is provided in S48 Table. We repeated these associations removing the top and bottom 30 individuals with the most extreme dfbeta values. This suggested an increase in the magnitude of the association of PI-MZ with FEV1 and height.

It was noted on examination of the residual versus fitted plots from the fixed effects meta-analyses that when FEV1 or $(\text{FEV1/FVC ratio})^3$ were analysed, on some occasions there was a bias in the residuals from individuals in ELSA who have COPD (i.e. a very small number of individuals, but this could be detected from the plots). The basic analysis of PI-MZ vs PI-MM against FEV1 was repeated excluding ELSA individuals with COPD. The updated regression coefficient was 0.13 (95% CI: 0.08, 0.19) and was 0.07 (95% CI: 0.01, 0.12) for the height, height-squared adjusted model. We repeated the fixed effects analysis of PI-MZ vs PI-MM with height, excluding Boyd Orr due to the comparatively large effect estimate in this cohort. The regression coefficient was 1.45 cm (0.98cm, 1.92cm).

S1 Fig. EHH analysis of rs28929474 and rs17580 and neighbouring SNPs. A) Core haplotype involves rs28929474, rs1802959, rs28929471 and rs17580. B) Core haplotype involves rs1303, rs28929473, rs28929474, rs28929474, rs1802959, rs28929471, rs17580, rs28929472 and rs28929470.

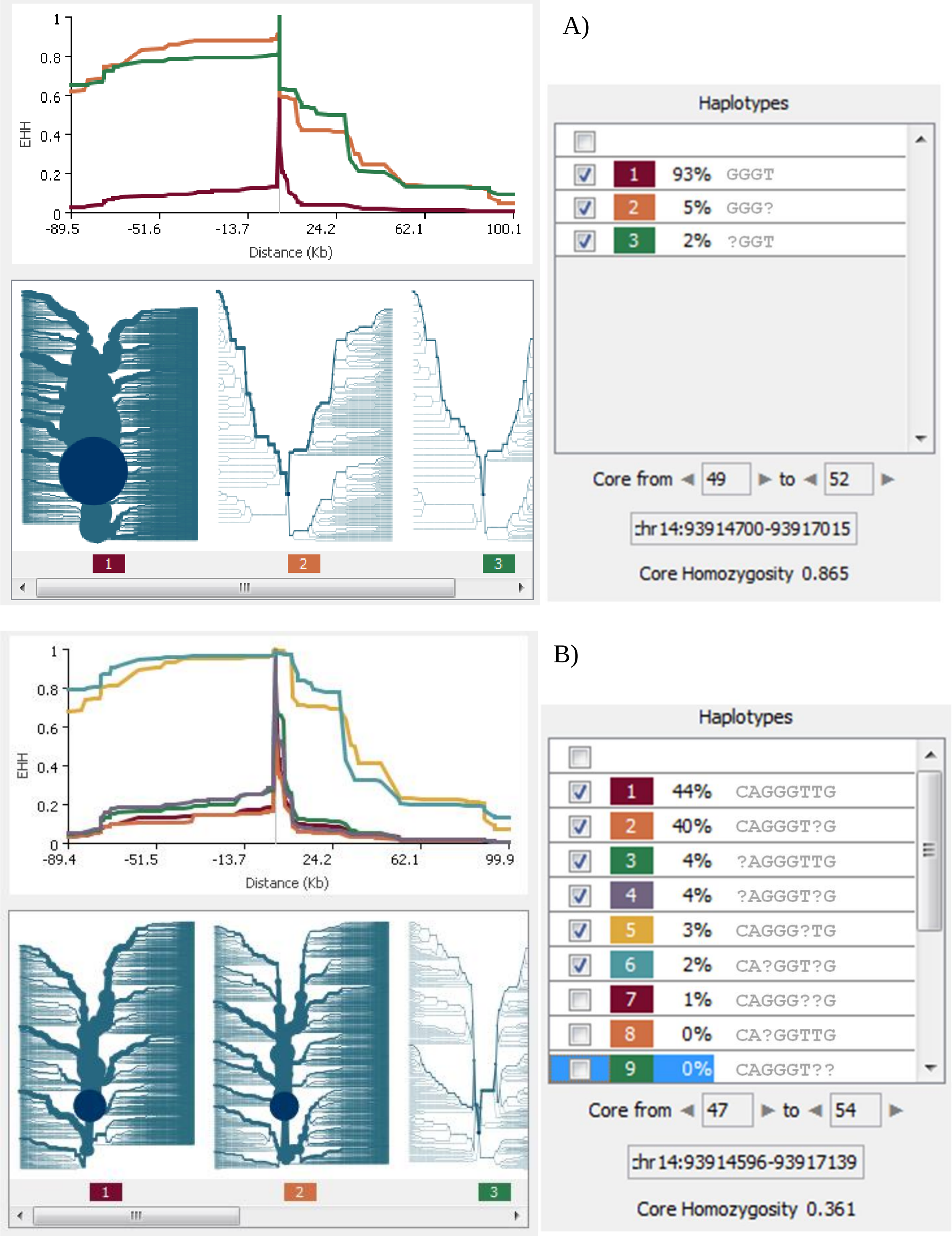

S2 Fig. Results observed from the web tool Haplotter http://haplotter.uchicago.edu/. Signatures of recent selection for the SERPINA1 gene and surrounding region (2Mb) have been tested using four different approaches: iHS, Fay and Wu's H, Tajima's D and F_{st}. According to the thresholds described in the literature for each test, there was no evidence of selection involving common SNPs in any case.

Gene name:SERPINA1 ID:5265

chromosome 14 [92834751:92845165]

-1 Mb | +1 Mb

iHS

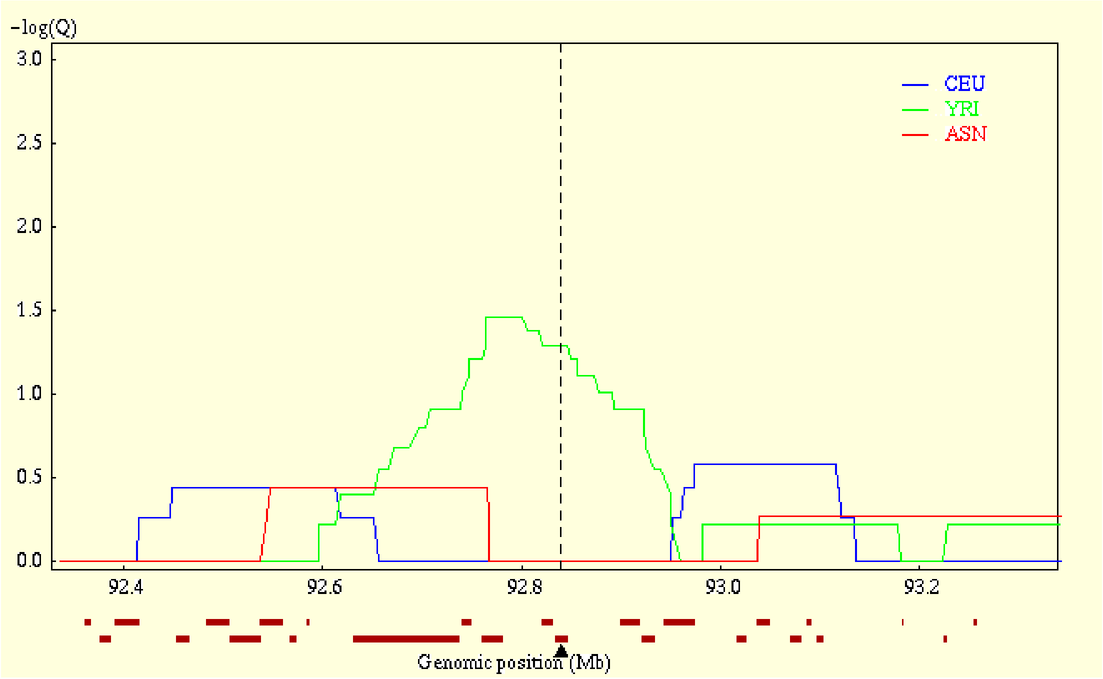

H

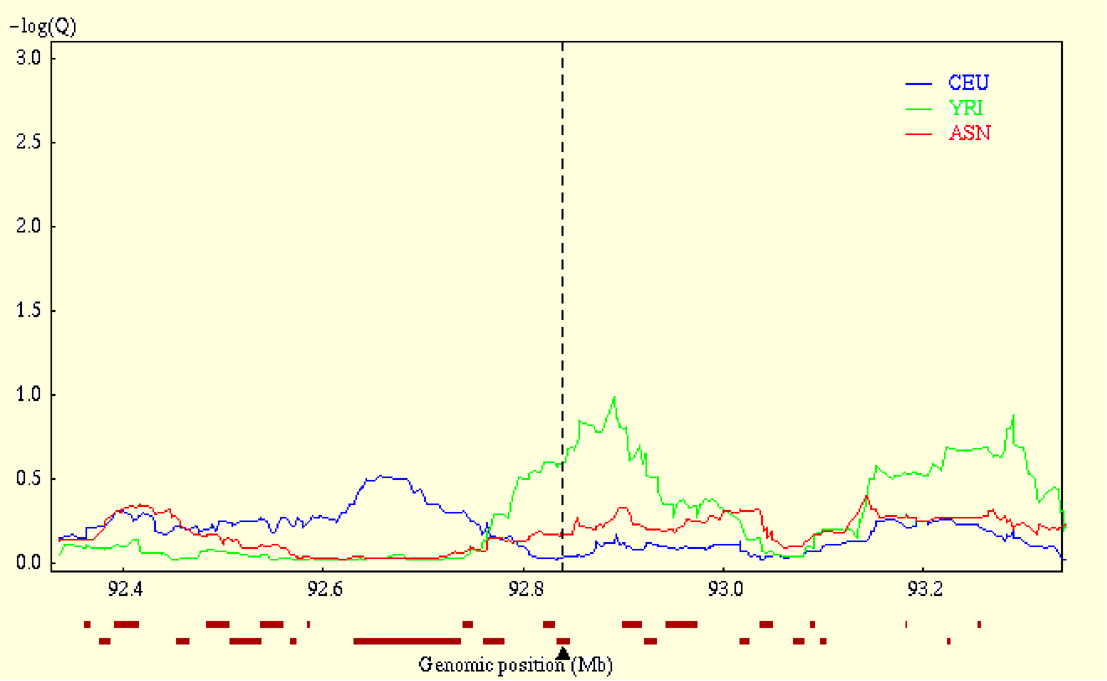

D

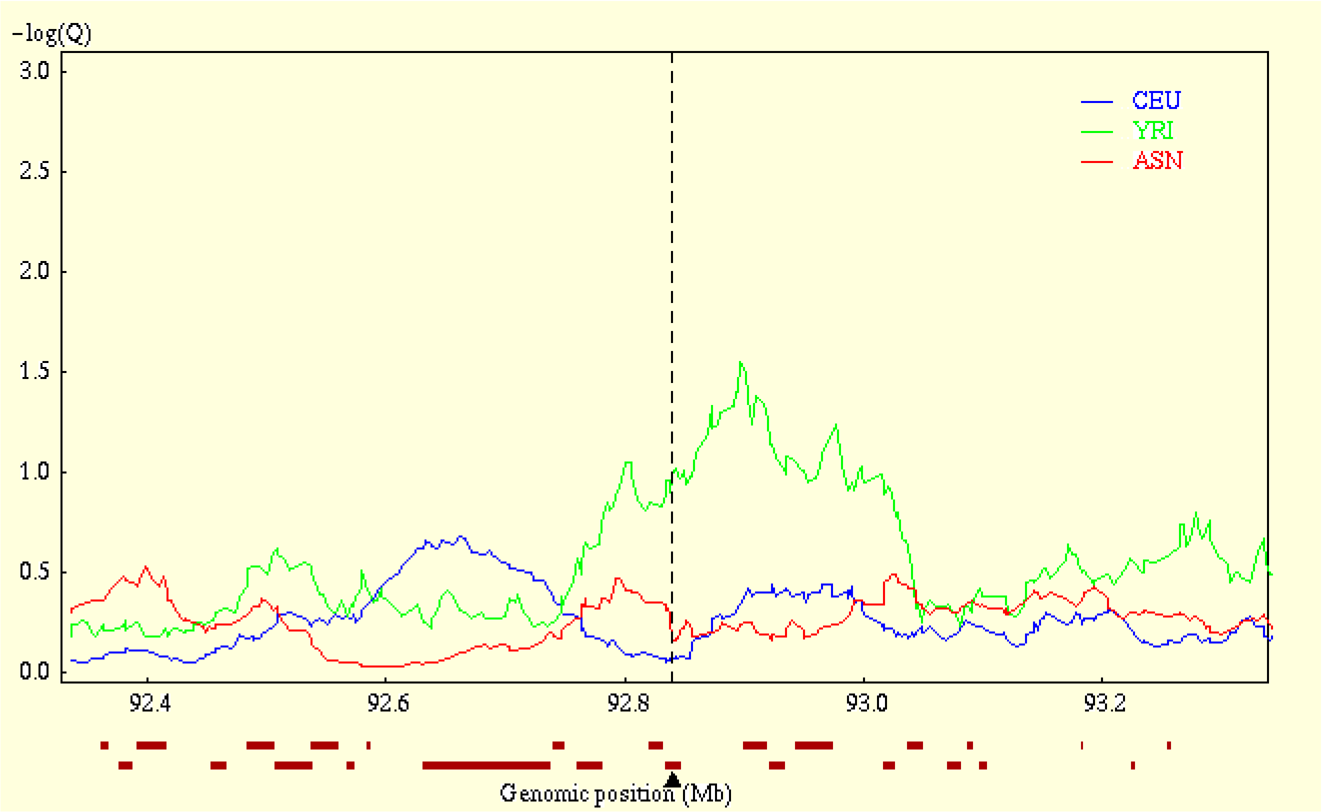
-log(Q)
3.0
2.5
2.0
1.5
1.0
0.5
0.0
CEU
YRI
ASN
92.4
92.6
92.8
93.0
93.2
Genomic position (Mb)

Fst

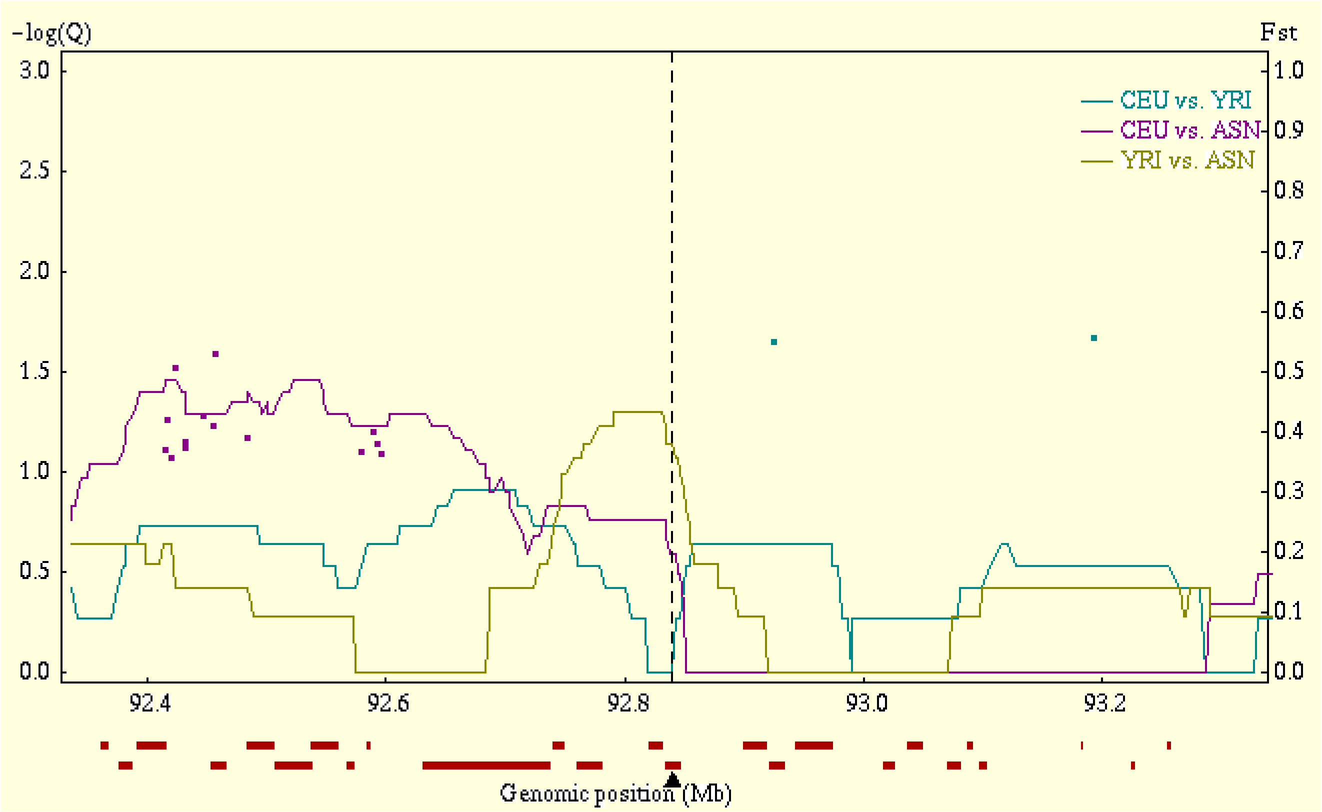
-log(Q)
Fst
3.0
2.5
2.0
1.5
1.0
0.5
0.0
1.0
0.9
0.8
0.7
0.6
0.5
0.4
0.3
0.2
0.1
0.0
CEU vs. YRI
CEU vs. ASN
YRI vs. ASN
92.4
92.6
92.8
93.0
93.2
Genomic position (Mb)

Genes in the region

Gene ID	Name	Region	CEU	YRI	ASN
90050	C14orf152	92375299 - 92385985	0.999955	0.999955	0.999954
51676	ASB2	92390554 - 92413808	0.999955	0.999955	0.999954
256369	C14orf48	92453683 - 92465250	0.351631	0.999955	0.999954
78990	OTUB2	92482765 - 92505317	0.351631	0.999955	0.999954
57062	DDX24	92507309 - 92537599	0.351631	0.999955	0.999954
122509	FAM14B	92537716 - 92559096	0.351631	0.999955	0.359281
3429	IFI27	92568017 - 92573073	0.351631	0.999955	0.359281
83982	FAM14A	92584162 - 92585964	0.351631	-	0.359281
57718	KIAA1622	92630690 - 92736113	0.999955	0.225666	0.359281
51156	SERPINA10	92739691 - 92749402	0.999955	0.063964	0.359281
866	SERPINA6	92760627 - 92779714	0.999955	0.036685	0.999954
390502	SERPINA2	92820692 - 92830320	0.999955	0.052676	0.999954
5265	SERPINA1	92834751 - 92845165	0.999955	0.052676	0.999954
256394	SERPINA11	92898844 - 92917877	0.999955	0.132766	0.999954
327657	SERPINA9	92921004 - 92932711	0.999955	0.170705	0.999954
145264	SERPINA12	92943661 - 92974222	0.541838	0.999955	0.999954
5267	SERPINA4	93017824 - 93026284	0.252749	0.607928	0.999954
5104	SERPINA5	93037852 - 93049493	0.252749	0.607928	0.539616
12	SERPINA3	93070812 - 93080432	0.252749	0.607928	0.539616
390503	LOC390503	93088276 - 93090906	0.252749	0.607928	0.539616
388007	SERPINA13	93097103 - 93103372	0.252749	0.607928	0.539616
145259	LAMR1P4	93182590 - 93183619	0.999955	0.999955	0.539616

S3 Fig. Estimation of the number of generations (n) computed from the proportion of haplotypes not recombined (X). Each point corresponds to a pairwise combination involving rs28929474 and neighbouring SNPs at a distance ranging from 104 bp to 224 Kb.

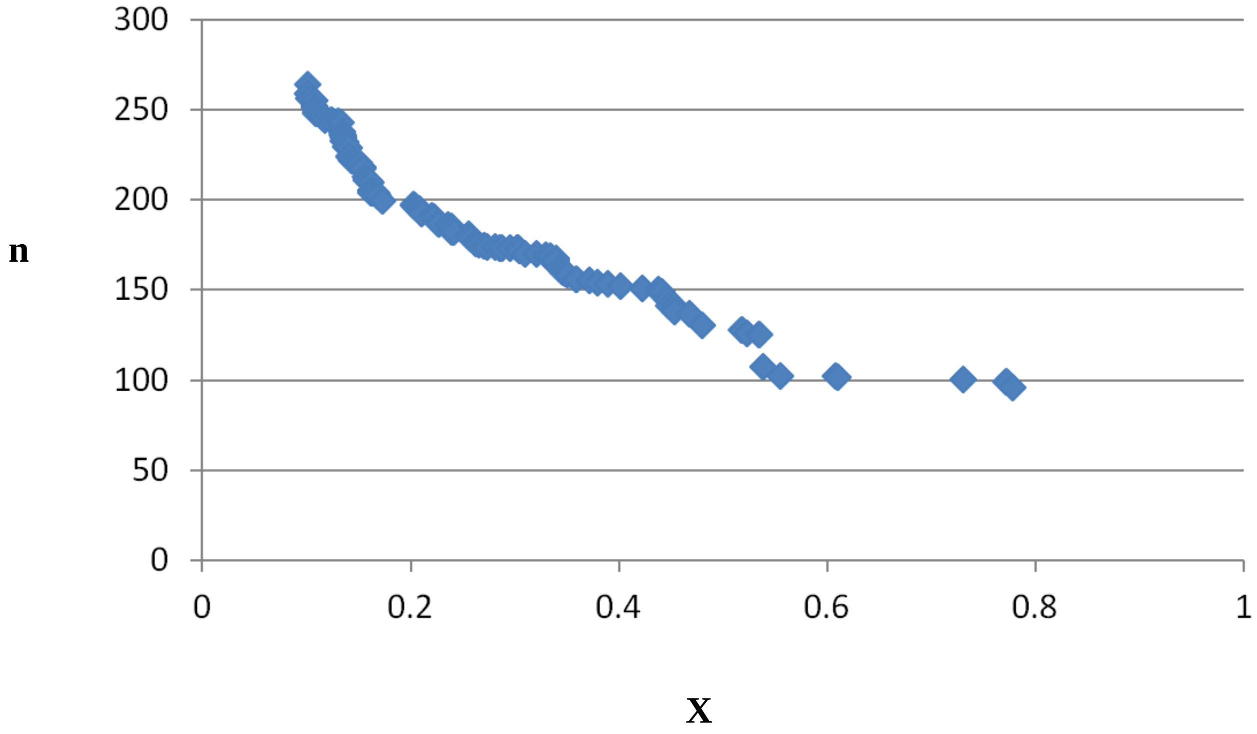

S1 Table. Genotyping Quality

Analysis	Cohort	SNP	Call rate (%)	Duplicate concordance rate (%)
Alpha 1-antitrypsin deficiency	BO	rs28929474	99.9	100
	CaPS		99.2	100
	ELSA		98.8	100
	LBC1921		98.6	100
	HAS		94.0	Not available
	HCS		98.1	Not available
	NSHD		N/A	N/A
	WHII		99.1	100
	BO	rs17580	97.2	100
	CaPS		94.5	100
	ELSA		98.0	99.9
	LBC1921		96.3	100
	HAS/HCS		98.1	Not available
	NSHD		N/A	N/A

	WHII		99.2	100
Cystic fibrosis	BO	rs113993960	99.6	100
	CaPS		98.3	100
	ELSA		99.8	100
	LBC1921		98.3	100
	HAS/HCS		98.4	Not available
	NSHD		99.0	100
	WHII		99.4	100
Phenylketonuria	BO	rs5030858	99.5	100
	CaPS		99.7	100
	ELSA		99.2	100
	LBC1921		99.3	100
	HAS/HCS		98.5	Not available
	NSHD		99.2	100
	WHII		99.1	100
	BO	rs5030861	99.9	100
	CaPS		99.6	100

	ELSA		99.2	100
	LBC1921		98.6	100
	HAS/HCS		97.8	Not available
	NSHD		99.0	N/A
	WHII		98.6	100
	BO	rs75193786	99.2	100
	CaPS		98.4	100
	ELSA		99.4	100
	LBC1921		98.6	100
	HAS/HCS		97.5	Not available
	NSHD		97.8	N/A
	WHII		99.1	100
Medium chain acyl-coA dehydrogenase deficiency	BO	rs77931234	99.6	100
	CaPS		98.8	100
	ELSA		99.4	100

	LBC1921		97.0	100
	HAS/HCS		97.8	Not available
	NSHD		98.9	100
	WHII		98.4	100

S2 Table. Sample size by analysis

Outcome	Covariates	Genetic exposure	Number of carriers	Number of non-carriers	Total sample size analysed
weight	age,sex	PI-MS vs PI-MM	1746	15873	17619
height	age,sex	PI-MS vs PI-MM	1739	15902	17641
BMI	age,sex	PI-MS vs PI-MM	1732	15782	17514
FEV1	age,sex	PI-MS vs PI-MM	1490	13721	15211
FVC	age,sex	PI-MS vs PI-MM	1490	13721	15211
FEV1/FVC ratio	age,sex	PI-MS vs PI-MM	1490	13721	15211
FEV1	age,sex, hadj	PI-MS vs PI-MM	1471	13579	15050
FVC	age,sex, hadj	PI-MS vs PI-MM	1471	13579	15050
FEV1/FVC ratio	age,sex, hadj	PI-MS vs PI-MM	1471	13579	15050
FEV1	age,sex, hadj3	PI-MS vs PI-MM	1471	13579	15050
FVC	age,sex, hadj3	PI-MS vs PI-MM	1471	13579	15050
FEV1/FVC ratio	age,sex, hadj3	PI-MS vs PI-MM	1471	13579	15050
COPD	age,sex	PI-MS vs PI-MM	1471	13579	15050
height	age,sex - lfadj	PI-MS vs PI-MM	1471	13579	15050
grip strength	age,sex	PI-MS vs PI-MM	1114	10025	11139
walk speed	age,sex	PI-MS vs PI-MM	1073	10088	11161
ability to balance	age,sex	PI-MS vs PI-MM	994	9209	10203
chair rise speed	age,sex	PI-MS vs PI-MM	804	7547	8351
height	age,sex	PI-MZ vs PI-MM	725	15902	16627
weight	age,sex	PI-MZ vs PI-MM	725	15873	16598
height_trim	age,sex	PI-MZ vs PI-MM	724	15871	16595
BMI	age,sex	PI-MZ vs PI-MM	722	15782	16504
weight_trim	age,sex	PI-MZ vs PI-MM	719	15763	16482
FEV1	exsmok	PI-MS vs PI-MM	665	6135	6800
FVC	exsmok	PI-MS vs PI-MM	665	6135	6800
FEV1/FVC ratio	exsmok	PI-MS vs PI-MM	665	6135	6800
FEV1	age,sex	PI-MZ vs PI-MM	640	13721	14361
FEV1_trim	age,sex	PI-MZ vs PI-MM	640	13697	14337
FVC	age,sex	PI-MZ vs PI-MM	640	13721	14361
FEV1/FVC ratio	age,sex	PI-MZ vs PI-MM	640	13721	14361
FVC_trim	age,sex	PI-MZ vs PI-MM	637	13682	14319
FEV1	age,sex, hadj	PI-MZ vs PI-MM	633	13579	14212
FEV1_trim	age,sex, hadj	PI-MZ vs PI-MM	633	13556	14189
FVC	age,sex, hadj	PI-MZ vs PI-MM	633	13579	14212
FEV1/FVC ratio	age,sex, hadj	PI-MZ vs PI-MM	633	13579	14212
FEV1	age,sex, hadj3	PI-MZ vs PI-MM	633	13579	14212
FVC	age,sex, hadj3	PI-MZ vs PI-MM	633	13579	14212
FEV1/FVC ratio	age,sex, hadj3	PI-MZ vs PI-MM	633	13579	14212
COPD	age,sex	PI-MZ vs PI-MM	633	13579	14212
height	age,sex - lfadj	PI-MZ vs PI-MM	633	13579	14212
height_trim	age,sex - lfadj	PI-MZ vs PI-MM	632	13555	14187
FVC_trim	age,sex, hadj	PI-MZ vs PI-MM	630	13540	14170
FEV1	neversmok	PI-MS vs PI-MM	558	5219	5777
FVC	neversmok	PI-MS vs PI-MM	558	5219	5777
FEV1/FVC ratio	neversmok	PI-MS vs PI-MM	558	5219	5777
TUG speed	age,sex	PI-MS vs PI-MM	481	4381	4862
grip strength	age,sex	PI-MZ vs PI-MM	467	10025	10492
FEV1	age,sex	DeltaF508 carrier	452	14854	15306
FVC	age,sex	DeltaF508 carrier	452	14854	15306
FEV1/FVC ratio	age,sex	DeltaF508 carrier	452	14854	15306
walk speed	age,sex	PI-MZ vs PI-MM	450	10088	10538
FEV1	hadj	DeltaF508 carrier	442	14697	15139
FVC	hadj	DeltaF508 carrier	442	14697	15139
FEV1/FVC ratio	hadj	DeltaF508 carrier	442	14697	15139
COPD	age,sex	DeltaF508 carrier	442	14697	15139
ability to balance	age,sex	PI-MZ vs PI-MM	422	9209	9631
chair rise speed	age,sex	PI-MZ vs PI-MM	348	7547	7895
FEV1	exsmok	PI-MZ vs PI-MM	287	6135	6422
FVC	exsmok	PI-MZ vs PI-MM	287	6135	6422
FEV1/FVC ratio	exsmok	PI-MZ vs PI-MM	287	6135	6422
FEV1	currsmok	PI-MS vs PI-MM	263	2333	2596
FVC	currsmok	PI-MS vs PI-MM	263	2333	2596
FEV1/FVC ratio	currsmok	PI-MS vs PI-MM	263	2333	2596
FEV1	neversmok	PI-MZ vs PI-MM	254	5219	5473
FVC	neversmok	PI-MZ vs PI-MM	254	5219	5473

FEV1/FVC ratio	neversmok	PI-MZ vs PI-MM	254	5219	5473
FEV1	exsmok	DeltaF508 carrier	221	6656	6877
FVC	exsmok	DeltaF508 carrier	221	6656	6877
FEV1/FVC ratio	exsmok	DeltaF508 carrier	221	6656	6877
TUG speed	age,sex	PI-MZ vs PI-MM	218	4381	4599
semantic fluency	age,sex	K304E carrier	208	12753	12961
height	age,sex - under 55	PI-MZ vs PI-MM	204	4348	4552
word recall ability	age,sex	K304E carrier	193	11505	11698
walk speed	age,sex	K304E carrier	177	10541	10718
grip strength	age,sex	K304E carrier	171	11651	11822
ability to balance	age,sex	K304E carrier	156	10650	10806
FEV1	neversmok	DeltaF508 carrier	151	5603	5754
FVC	neversmok	DeltaF508 carrier	151	5603	5754
FEV1/FVC ratio	neversmok	DeltaF508 carrier	151	5603	5754
chair rise speed	age,sex	K304E carrier	134	8720	8854
FEV1	age,sex, in COPD	PI-MS vs PI-MM	118	1118	1236
FEV1	age,sex,hadj,SSadj in COPD	PI-MS vs PI-MM	118	1116	1234
FVC	age,sex, in COPD	PI-MS vs PI-MM	118	1118	1236
FVC	age,sex,hadj,SSadj in COPD	PI-MS vs PI-MM	118	1116	1234
FEV1/FVC ratio	age,sex, in COPD	PI-MS vs PI-MM	118	1118	1236
FEV1/FVC ratio	age,sex,hadj,SSadj in COPD	PI-MS vs PI-MM	118	1116	1234
FEV1	currsmok	PI-MZ vs PI-MM	97	2333	2430
FVC	currsmok	PI-MZ vs PI-MM	97	2333	2430
FEV1/FVC ratio	currsmok	PI-MZ vs PI-MM	97	2333	2430
AH4	age,sex	K304E carrier	94	5232	5326
semantic fluency	age,sex	PAH mutation carrier	81	12708	12789
FEV1	currsmok	DeltaF508 carrier	79	2559	2638
FVC	currsmok	DeltaF508 carrier	79	2559	2638
FEV1/FVC ratio	currsmok	DeltaF508 carrier	79	2559	2638
word recall ability	age,sex	PAH mutation carrier	74	11456	11530
TUG speed	age,sex	K304E carrier	69	5053	5122
grip strength	age,sex	PAH mutation carrier	69	11587	11656
walk speed	age,sex	PAH mutation carrier	67	10525	10592
ability to balance	age,sex	PAH mutation carrier	62	10589	10651
chair rise speed	age,sex	PAH mutation carrier	54	8661	8715
FEV1	age,sex, in COPD	PI-MZ vs PI-MM	51	1118	1169
FEV1	age,sex,hadj,SSadj in COPD	PI-MZ vs PI-MM	51	1116	1167
FVC	age,sex, in COPD	PI-MZ vs PI-MM	51	1118	1169
FVC	age,sex,hadj,SSadj in COPD	PI-MZ vs PI-MM	51	1116	1167
FEV1/FVC ratio	age,sex, in COPD	PI-MZ vs PI-MM	51	1118	1169
FEV1/FVC ratio	age,sex,hadj,SSadj in COPD	PI-MZ vs PI-MM	51	1116	1167
height	age,sex	PI-SZ vs PI-MM	42	15902	15944
FEV1	COPD	DeltaF508 carrier	41	1212	1253
FVC	COPD	DeltaF508 carrier	41	1212	1253
FEV1/FVC ratio	COPD	DeltaF508 carrier	41	1212	1253
height	age,sex	PI-SS vs PI-MM	41	15902	15943
weight	age,sex	PI-SZ vs PI-MM	41	15873	15914
weight	age,sex	PI-SS vs PI-MM	41	15873	15914
BMI	age,sex	PI-SZ vs PI-MM	41	15782	15823
BMI	age,sex	PI-SS vs PI-MM	41	15782	15823
AH4	age,sex	PAH mutation carrier	38	5225	5263
FEV1	age,sex, hadj	PI-SZ vs PI-MM	37	13579	13616
FEV1	age,sex, hadj	PI-SS vs PI-MM	37	13579	13616
FEV1	age,sex	PI-SZ vs PI-MM	37	13721	13758
FVC	age,sex	PI-SZ vs PI-MM	37	13721	13758
FEV1/FVC ratio	age,sex	PI-SZ vs PI-MM	37	13721	13758
FVC	age,sex, hadj	PI-SZ vs PI-MM	37	13579	13616
FEV1/FVC ratio	age,sex, hadj	PI-SZ vs PI-MM	37	13579	13616

FEV1	age,sex, hadj3	PI-SZ vs PI-MM	37	13579	13616
FVC	age,sex, hadj3	PI-SZ vs PI-MM	37	13579	13616
FEV1/FVC ratio	age,sex, hadj3	PI-SZ vs PI-MM	37	13579	13616
height	age,sex - lfadj	PI-SZ vs PI-MM	37	13579	13616
height	age,sex - lfadj	PI-SS vs PI-MM	37	13579	13616
FEV1	age,sex	PI-SS vs PI-MM	37	13721	13758
FVC	age,sex	PI-SS vs PI-MM	37	13721	13758
FEV1/FVC ratio	age,sex	PI-SS vs PI-MM	37	13721	13758
FVC	age,sex, hadj	PI-SS vs PI-MM	37	13579	13616
FEV1/FVC ratio	age,sex, hadj	PI-SS vs PI-MM	37	13579	13616
FEV1	age,sex, hadj3	PI-SS vs PI-MM	37	13579	13616
FVC	age,sex, hadj3	PI-SS vs PI-MM	37	13579	13616
FEV1/FVC ratio	age,sex, hadj3	PI-SS vs PI-MM	37	13579	13616
walk speed	age,sex	PI-SS vs PI-MM	32	10088	10120
grip strength	age,sex	PI-SZ vs PI-MM	31	10025	10056
TUG speed	age,sex	PAH mutation carrier	30	5028	5058
NART	age,sex	PAH mutation carrier	29	4203	4232
walk speed	age,sex	PI-SZ vs PI-MM	28	10088	10116
grip strength	age,sex	PI-SS vs PI-MM	28	10025	10053
chair rise speed	age,sex	PI-SZ vs PI-MM	25	7547	7572
chair rise speed	age,sex	PI-SS vs PI-MM	19	7547	7566
FEV1	exsmok	PI-SZ vs PI-MM	18	6135	6153
FVC	exsmok	PI-SZ vs PI-MM	18	6135	6153
FEV1/FVC ratio	exsmok	PI-SZ vs PI-MM	18	6135	6153
FEV1	neversmok	PI-SZ vs PI-MM	17	5219	5236
FVC	neversmok	PI-SZ vs PI-MM	17	5219	5236
FEV1/FVC ratio	neversmok	PI-SZ vs PI-MM	17	5219	5236
FEV1	exsmok	PI-SS vs PI-MM	17	6135	6152
FVC	exsmok	PI-SS vs PI-MM	17	6135	6152
FEV1/FVC ratio	exsmok	PI-SS vs PI-MM	17	6135	6152
FEV1	neversmok	PI-SS vs PI-MM	16	5219	5235
FVC	neversmok	PI-SS vs PI-MM	16	5219	5235
FEV1/FVC ratio	neversmok	PI-SS vs PI-MM	16	5219	5235
TUG speed	age,sex	PI-SZ vs PI-MM	14	4381	4395
TUG speed	age,sex	PI-SS vs PI-MM	12	4381	4393
height	age,sex	PI-ZZ vs PI-MM	8	15902	15910
weight	age,sex	PI-ZZ vs PI-MM	8	15873	15881
BMI	age,sex	PI-ZZ vs PI-MM	8	15782	15790
FEV1	age,sex, hadj	PI-ZZ vs PI-MM	6	13579	13585
FEV1	age,sex	PI-ZZ vs PI-MM	6	13721	13727
FVC	age,sex	PI-ZZ vs PI-MM	6	13721	13727
height	age,sex - lfadj	PI-ZZ vs PI-MM	6	13579	13585
FEV1/FVC ratio	age,sex	PI-ZZ vs PI-MM	6	13721	13727
FVC	age,sex, hadj	PI-ZZ vs PI-MM	6	13579	13585
FEV1/FVC ratio	age,sex, hadj	PI-ZZ vs PI-MM	6	13579	13585
FEV1	age,sex, hadj3	PI-ZZ vs PI-MM	6	13579	13585
FVC	age,sex, hadj3	PI-ZZ vs PI-MM	6	13579	13585
FEV1/FVC ratio	age,sex, hadj3	PI-ZZ vs PI-MM	6	13579	13585
FEV1	currsmok	PI-SS vs PI-MM	4	2333	2337
FVC	currsmok	PI-SS vs PI-MM	4	2333	2337
FEV1/FVC ratio	currsmok	PI-SS vs PI-MM	4	2333	2337
FEV1	neversmok	PI-ZZ vs PI-MM	3	5219	5222
FVC	neversmok	PI-ZZ vs PI-MM	3	5219	5222
FEV1/FVC ratio	neversmok	PI-ZZ vs PI-MM	3	5219	5222

Hadj – height + height^2 adjusted
Hadj3 – height + height^2 + height^3 adjusted
SSadj – smoking status adjusted
Lfadj – FEV1 and FVC adjusted
Exsmok – analysis in ex smokers
Neversmok-analysis in never smokers
Currsmok – analysis in current smokers
COPD – analysis in individuals classed as having COPD

Alpha 1-antitrypsin deficiency

S3 Table. Alpha 1-antitrypsin deficiency PI status frequencies

Cohort	MM	MS	MZ	SS	SZ	ZZ	Total, N[a]	% female	Mean age[b] (SE)
BO	232	24	6	0	0	0	262	54.96	69.61(0.26)
CaPS	1112	112	61	0	3	1	1289	0	53.79(0.14)
ELSA	4279	450	195	11	16	1	4952	53.92	65.59(0.13)
HAS	155	15	13	1	2	0	186	35.48	76.38(0.17)
HCS	2133	256	111	6	7	0	2513	47	66.09(0.06)
LBC1921	443	40	17	3	1	0	504	58.73	79.06(0.03)
NSHD	1704	191	81	6	2	1	1985	52.34	53.45(0.00)
WHII	3663	402	156	10	6	3	4240	29.41	60.74(0.09)
Combined	13721	1490	640	37	37	6	15931	41.7	62.53(0.07)
Combined (%)	86.13	9.35	4.02	0.23	0.23	0.04	100		

[a] Total number of individuals is total number with a valid PI status, sex, age and lung function
[b] mean age at wave of lung function outcomes

S4 Table. HWE of PI-S (rs17580)

Cohort	T/T	T/A	A/A	Total	HWE P-value
BO	238	24	0	262	0.437
CaPS	1174	115	0	1289	0.094
ELSA	4475	466	11	4952	0.756
HAS	168	17	1	186	0.436
HCS	2244	263	6	2513	0.557
LBC1921	460	41	3	504	0.056
NSHD[a]	1786	193	6	1985	0.746
WHII	3822	408	10	4240	0.797
TOTAL	14367	1527	37	15931	0.593

Based on all individuals with a valid PI status, age, sex and lung function outcomes

[a] Derived from PI classes from isoelectric focusing(17)

S5 Table. HWE of PI-Z (rs28929474)

Cohort	G/G	G/A	A/A	Total	HWE P-value
BO	256	6	0	262	0.851
CaPS	1224	64	1	1289	0.862
ELSA	4740	211	1	4952	0.384
HAS	171	15	0	186	0.567
HCS	2395	118	0	2513	0.228
LBC1921	486	18	0	504	0.683
NSHD[a]	1901	83	1	1985	0.923
WHII	4075	162	3	4240	0.291
TOTAL	15248	677	6	15931	0.587

Based on all individuals with a valid PI status, age, sex and lung function outcomes

[a] Derived from PI classes from isoelectric focusing(17)

S6 Table. Association of PI status with lung function, adjusted for age and sex

Outcome	Cohort	Regression Coefficient (95% CI)				
		MS VS. MM	MZ VS. MM	SS VS. MM	SZ VS. MM	ZZ VS. MM
Maximum FEV1	BO	-0.05(-0.36,0.27)	0.45(-0.14,1.04)	[a]		
	CaPS	0.07(-0.10,0.24)	0.11(-0.12,0.33)			
	ELSA	0.05(-0.03,0.12)	0.12*(0.01,0.23)			
	HAS	-0.49*(-0.94,-0.05)	0.08(-0.39,0.55)			
	HCS	-0.01(-0.10,0.09)	0.05(-0.10,0.19)			
	LBC1921	-0.06(-0.31,0.19)	0.16(-0.22,0.54)			
	NSHD	0.02(-0.09,0.13)	0.18*(0.01,0.35)			
	WHII	0.05(-0.03,0.12)	0.18**(0.06,0.29)			
	Combined Fixed Effect	0.03(-0.01,0.07)	0.13****(0.07,0.19)	0.01(-0.23,0.26)	0.06(-0.18,0.31)	-0.29(-0.89,0.32)
	Combined Random Effect	0.03(-0.01,0.07)	0.13****(0.07,0.19)	0.02(-0.22,0.26)	0.07(-0.18,0.31)	-0.35(-0.95,0.25)
	Estimated variance of random effect	1.20e-13(0.00e+00,.)	1.09e-19(0.00e+00,.)	7.37e-12(4.15e-26,1.31e+03)	2.04e-14(5.46e-28,7.65e-01)	5.65e-10(0.00e+00,.)
Maximum FVC	BO	0.03(-0.26,0.32)	0.43(-0.11,0.97)	[a]		
	CaPS	0.08(-0.10,0.26)	0.15(-0.09,0.39)			
	ELSA	0.02(-0.05,0.09)	0.12*(0.01,0.22)			
	HAS	-0.35(-0.73,0.03)	0.33(-0.09,0.74)			
	HCS	-0.02(-0.11,0.07)	0.14*(0.01,0.27)			
	LBC1921	-0.07(-0.32,0.17)	0.20(-0.17,0.56)			
	NSHD	0.02(-0.09,0.13)	0.20*(0.04,0.37)			
	WHII	-0.01(-0.08,0.07)	0.19**(0.07,0.30)			
	Combined Fixed Effect	0.01(-0.03,0.04)	0.16****(0.10,0.22)	-0.12(-0.35,0.12)	0.07(-0.17,0.31)	-0.13(-0.71,0.46)
	Combined Random Effect	0.00(-0.04,0.04)	0.16****(0.10,0.22)	-0.11(-0.34,0.12)	0.07(-0.17,0.30)	-0.18(-0.77,0.41)
	Estimated variance of random effect	3.76e-15(1.01e-28,1.40e-01)	3.53e-19(0.00e+00,.)	4.07e-14(8.72e-29,1.90e+01)	9.77e-13(1.11e-31,8.59e+06)	3.87e-14(2.43e-32,6.18e+04)

FEV1/FVC ratio	BO	-0.31(-0.73,0.11)	0.15(-0.64,0.95)	[a]		
	CaPS	0.02(-0.15,0.18)	0.03(-0.20,0.25)			
	ELSA	0.04(-0.06,0.13)	0.01(-0.14,0.15)			
	HAS	-0.34(-0.89,0.22)	-0.29(-0.85,0.27)			
	HCS	0.03(-0.10,0.15)	-0.13(-0.32,0.06)			
	LBC1921	-0.02(-0.34,0.31)	-0.15(-0.65,0.34)			
	NSHD	-0.03(-0.18,0.12)	-0.10(-0.32,0.12)			
	WHII	0.11*(0.01,0.21)	0.02(-0.14,0.18)			
	Combined Fixed Effect	0.03(-0.02,0.09)	-0.04(-0.11,0.04)	0.23(-0.09,0.55)	0.01(-0.31,0.32)	-0.69(-1.47,0.10)
	Combined Random Effect	0.03(-0.02,0.09)	-0.03(-0.11,0.04)	0.23(-0.09,0.55)	0.01(-0.31,0.32)	-0.55(-1.43,0.34)
	Estimated variance of random effect	3.41e-17(4.23e-30,2.75e-04)	1.34e-18(9.21e-33,1.95e-04)	4.77e-17(7.55e-32,3.02e-02)	2.73e-15(2.55e-32,2.92e+02)	1.60e-01(2.99e-04,8.50e+01)

Outcomes z-scored within cohorts

*p<0.05, **p<0.01, ***p<0.001, ****p<0.0001

[a] Cohort specific estimates are suppressed for PI-SS, SZ and ZZ due to the small number of carriers

S7 Table. Association of PI status with transformed lung function, adjusted for age and sex

Outcome	Cohort	Regression Coefficient (95% CI)				
		MS VS. MM	MZ VS. MM	SS VS. MM	SZ VS. MM	ZZ VS. MM
Sqrt(Maximum FVC)	BO	-0.02(-0.32,0.27)	0.43(-0.12,0.97)	[a]		
	CaPS	0.08(-0.10,0.25)	0.13(-0.10,0.36)			
	ELSA	0.02(-0.05,0.09)	0.10(-0.00,0.21)			
	HAS	-0.35(-0.73,0.03)	0.31(-0.10,0.72)			
	HCS	-0.01(-0.10,0.07)	0.12(-0.00,0.25)			
	LBC1921	-0.08(-0.33,0.17)	0.18(-0.19,0.55)			
	NSHD	0.02(-0.09,0.13)	0.20*(0.04,0.37)			
	WHII	-0.01(-0.08,0.06)	0.18**(0.07,0.29)			
	Combined Fixed Effect	0.00(-0.04,0.04)	0.15****(0.09,0.21)	-0.11(-0.34,0.13)	0.07(-0.16,0.31)	-0.11(-0.69,0.46)
	Combined Random Effect	0.00(-0.04,0.04)	0.15****(0.09,0.21)	-0.10(-0.33,0.13)	0.07(-0.16,0.31)	-0.17(-0.75,0.42)
	Estimated variance of random effect	1.03e-14(5.17e-28,2.07e-01)	4.11e-17(9.66e-31,1.75e-03)	1.13e-14(1.77e-30,7.23e+01)	1.80e-12(0.00e+00,.)	4.18e-09(3.76e-27,4.66e+09)
$(FEV1/FVC\ ratio)^3$	BO	-0.30(-0.72,0.13)	0.07(-0.75,0.88)	[a]		
	CaPS	-0.01(-0.18,0.17)	0.02(-0.22,0.25)			
	ELSA	0.03(-0.07,0.12)	0.02(-0.12,0.16)			
	HAS	-0.27(-0.81,0.27)	-0.37(-0.94,0.19)			
	HCS	0.02(-0.11,0.14)	-0.08(-0.27,0.11)			
	LBC1921	-0.06(-0.38,0.26)	-0.14(-0.64,0.35)			
	NSHD	-0.03(-0.18,0.12)	-0.14(-0.36,0.08)			
	WHII	0.10*(0.00,0.21)	0.03(-0.13,0.19)			
	Combined Fixed Effect	0.03(-0.03,0.08)	-0.03(-0.11,0.05)	0.19(-0.13,0.50)	-0.07(-0.39,0.25)	-0.61(-1.40,0.17)
	Combined Random Effect	0.03(-0.03,0.08)	-0.03(-0.10,0.05)	0.19(-0.13,0.50)	-0.07(-0.38,0.25)	-0.57(-1.35,0.21)
	Estimated variance of random effect	1.74e-17(5.35e-31,5.65e-04)	2.18e-17(2.82e-30,1.69e-04)	8.96e-20(7.43e-34,1.08e-05)	4.48e-06(3.06e-19,6.56e+07)	7.56e-10(3.04e-32,1.88e+13)

Outcomes z-scored within cohorts
*p<0.05, **p<0.01, ***p<0.001, ****p<0.0001
[a] Cohort specific estimates are suppressed for PI-SS, SZ and ZZ due to the small number of carriers

S8 Table. Association of PI-MZ with lung function with +/-3SDs removed from outcome before standardisation, adjusted for age and sex

Outcome	Cohort	Regression Coefficient (95% CI)
Maximum FEV1	Combined Fixed Effect	0.14****(0.08,0.20)
	Combined Random Effect	0.14****(0.08,0.20)
	Estimated variance of random effect	2.19e-17(2.33e-29,2.05e-05)
Maximum FVC	Combined Fixed Effect	0.16****(0.10,0.22)
	Combined Random Effect	0.16****(0.10,0.22)
	Estimated variance of random effect	1.84e-17(9.28e-33,3.66e-02)

Outcomes z-scored within cohorts
*p<0.05, **p<0.01, ***p<0.001, ****p<0.0001

S9 Table. Association of PI-MZ with lung function, raw values without z-scoring, adjusted for age and sex

Outcome	Cohort	Regression Coefficient (95% CI) (Litres)
Maximum FEV1 (L)	BO	0.33(-0.10,0.75)
	CaPS	0.08(-0.09,0.25)
	ELSA	0.10*(0.01,0.19)
	HAS	0.05(-0.25,0.35)
	HCS	0.03(-0.07,0.13)
	LBC1921	0.10(-0.14,0.34)
	NSHD	0.12*(0.01,0.24)
	WHII	0.14**(0.05,0.23)
	Combined Fixed Effect	0.10****(0.06,0.15)
	Combined Random Effect	0.10****(0.05,0.14)
	Estimated variance of random effect	6.77e-21(1.15e-32,3.97e-09)
Maximum FVC (L)	BO	0.39(-0.10,0.89)
	CaPS	0.12(-0.07,0.30)
	ELSA	0.13*(0.02,0.24)
	HAS	0.26(-0.07,0.58)
	HCS	0.13*(0.01,0.25)
	LBC1921	0.14(-0.13,0.42)
	NSHD	0.18*(0.04,0.32)
	WHII	0.18**(0.07,0.29)
	Combined Fixed Effect	0.15****(0.10,0.21)
	Combined Random Effect	0.15****(0.10,0.21)
	Estimated variance of random effect	4.75e-21(5.59e-36,4.04e-06)
*p<0.05, **p<0.01, ***p<0.001, ****p<0.0001		

S10 Table. Association of PI status with lung function, adjusted for age and sex, in current smokers

Outcome	Cohort	Regression Coefficient (95% CI)		
		MS VS. MM	MZ VS. MM	SS VS. MM
Maximum FEV1	Combined Fixed Effect	0.04(-0.06,0.14)	0.09(-0.07,0.25)	-0.30(-1.08,0.48)
	Combined Random Effect	0.03(-0.07,0.13)	0.09(-0.07,0.25)	-0.31(-1.30,0.69)
	Estimated variance of random effect	1.58e-24(7.90e-45,3.15e-04)	7.96e-22(2.05e-41,3.10e-02)	5.09e-01(2.86e-02,9.04e+00)
Maximum FVC	Combined Fixed Effect	0.00(-0.09,0.10)	0.12(-0.04,0.28)	-0.66(-1.42,0.11)
	Combined Random Effect	-0.01(-0.11,0.08)	0.14(-0.02,0.29)	-0.67(-1.69,0.35)
	Estimated variance of random effect	8.57e-23(1.06e-47,6.95e+02)	3.94e-22[a]	5.89e-01(4.30e-02,8.08e+00)
FEV1/FVC ratio	Combined Fixed Effect	0.07(-0.07,0.20)	-0.06(-0.28,0.15)	0.77(-0.29,1.83)
	Combined Random Effect	0.06(-0.07,0.19)	-0.02(-0.23,0.18)	0.74(-0.28,1.76)
	Estimated variance of random effect	2.75e-11(0.00e+00,.)	1.42e-13(5.60e-33,3.59e+06)	9.39e-20(5.69e-39,1.55e+00)

Outcomes z-scored within cohorts
Cohort specific estimates are suppressed due to the small number of carriers
Meta-analysis not possible for PI-SZ and ZZ as there were fewer than three cohorts with adequate data
*p<0.05, **p<0.01, ***p<0.001, ****p<0.0001
[a] Standard error of the variance of the random effect not estimable

S11 Table. Association of PI status with transformed lung function, adjusted for age and sex, in current smokers

Outcome	Cohort	Regression Coefficient (95% CI)		
		MS VS. MM	MZ VS. MM	SS VS. MM
Sqrt(Maximum FVC)	Combined Fixed Effect	0.01(-0.09,0.10)	0.11(-0.04,0.27)	-0.72(-1.48,0.05)
	Combined Random Effect	-0.01(-0.11,0.08)	0.13(-0.02,0.29)	-0.70(-1.78,0.38)
	Estimated variance of random effect	1.05e-17(4.73e-36,2.33e+01)	4.69e-24(5.98e-42,3.67e-06)	6.98e-01(6.46e-02,7.55e+00)
$(FEV1/FVC\ ratio)^3$	Combined Fixed Effect	0.06(-0.07,0.19)	-0.03(-0.24,0.18)	0.81(-0.19,1.82)
	Combined Random Effect	0.05(-0.08,0.18)	-0.01(-0.21,0.19)	0.80(-0.21,1.81)
	Estimated variance of random effect	3.60e-15(5.28e-28,2.46e-02)	6.98e-16(7.32e-32,6.66e+00)	1.73e-12(2.54e-31,1.17e+07)

Outcomes z-scored within cohorts
Cohort specific estimates are suppressed due to the small number of carriers
Meta-analysis not possible for PI-SZ and ZZ as there were fewer than three cohorts with adequate data
*p<0.05, **p<0.01, ***p<0.001, ****p<0.0001

S1 Table. Association of PI status with lung function, adjusted for age and sex, in ex smokers

Outcome	Cohort	Regression Coefficient (95% CI)			
		MS VS. MM	MZ VS. MM	SS VS. MM	SZ VS. MM
Maximum FEV1	Combined Fixed Effect	-0.01(-0.07,0.05)	0.13**(0.04,0.22)	0.18(-0.18,0.54)	0.26(-0.09,0.61)
	Combined Random Effect	-0.00(-0.06,0.06)	0.15*(0.03,0.26)	0.21(-0.15,0.57)	NOCONVERGENCE[a]
	Estimated variance of random effect	1.85e-22(1.89e-36,1.81e-08)	6.60e-03(4.74e-04,9.19e-02)	3.72e-14(0.00e+00,.)	NOCONVERGENCE[a]
Maximum FVC	Combined Fixed Effect	-0.02(-0.08,0.03)	0.19****(0.11,0.28)	0.04(-0.31,0.39)	0.29(-0.04,0.63)
	Combined Random Effect	-0.02(-0.08,0.03)	0.20****(0.11,0.28)	0.06(-0.29,0.41)	0.28(-0.06,0.61)
	Estimated variance of random effect	8.94e-16(1.09e-29,7.31e-02)	3.94e-18(3.65e-34,4.25e-02)	9.69e-14(0.00e+00,.)	1.88e-12(4.93e-40,7.19e+15)
FEV1/FVC ratio	Combined Fixed Effect	0.04(-0.04,0.11)	-0.06(-0.17,0.05)	0.21(-0.25,0.67)	-0.06(-0.51,0.38)
	Combined Random Effect	0.04(-0.04,0.12)	-0.06(-0.19,0.08)	0.21(-0.25,0.68)	-0.06(-0.50,0.38)
	Estimated variance of random effect	6.41e-16(1.93e-30,2.14e-01)	7.46e-03(2.53e-04,2.19e-01)	4.70e-14(6.50e-32,3.39e+04)	8.66e-17(3.55e-35,2.11e+02)

Outcomes z-scored within cohorts
Cohort specific estimates are suppressed due to the small number of carriers
Meta-analysis not possible for PI- ZZ as there were fewer than three cohorts with adequate data
*p<0.05, **p<0.01, ***p<0.001, ****p<0.0001
[a] Random effects model failed to converge

S2 Table. Association of PI status with transformed lung function, adjusted for age and sex, in ex smokers

Outcome	Cohort	Regression Coefficient (95% CI)			
		MS VS. MM	MZ VS. MM	SS VS. MM	SZ VS. MM
Sqrt(Maximum FVC)	Combined Fixed Effect	-0.03(-0.08,0.03)	0.17****(0.08,0.25)	0.07(-0.27,0.41)	0.27(-0.06,0.60)
	Combined Random Effect	-0.03(-0.08,0.03)	0.17****(0.09,0.26)	0.10(-0.25,0.44)	0.26(-0.07,0.60)
	Estimated variance of random effect	2.89e-15(9.33e-30,8.94e-01)	5.26e-22(1.08e-39,2.57e-04)	1.01e-13(0.00e+00,.)	1.79e-14(3.31e-33,9.64e+04)
$(FEV1/FVC\ ratio)^3$	Combined Fixed Effect	0.04(-0.04,0.11)	-0.05(-0.17,0.06)	0.15(-0.31,0.61)	-0.14(-0.59,0.31)
	Combined Random Effect	0.04(-0.04,0.12)	-0.05(-0.19,0.08)	0.16(-0.31,0.63)	-0.14(-0.59,0.31)
	Estimated variance of random effect	6.21e-18(1.73e-33,2.23e-02)	5.30e-03(6.21e-05,4.52e-01)	4.43e-13(4.36e-29,4.51e+03)	9.95e-17(6.79e-33,1.46e+00)

Outcomes z-scored within cohorts
Cohort specific estimates are suppressed due to the small number of carriers
Meta-analysis not possible for PI- ZZ as there were fewer than three cohorts with adequate data
*p<0.05, **p<0.01, ***p<0.001, ****p<0.0001

S3 Table. Association of PI status with lung function, adjusted for age and sex, in never smokers

Outcome	Cohort	Regression Coefficient (95% CI)				
		MS VS. MM	MZ VS. MM	SS VS. MM	SZ VS. MM	ZZ VS. MM
Maximum FEV1	Combined Fixed Effect	0.07*(0.01,0.13)	0.12**(0.03,0.21)	-0.11(-0.45,0.24)	-0.17(-0.51,0.17)	0.41(-0.39,1.22)
	Combined Random Effect	0.07*(0.01,0.13)	0.11*(0.03,0.20)	-0.11(-0.46,0.23)	-0.14(-0.48,0.19)	0.40(-0.44,1.25)
	Estimated variance of random effect	7.06e-20(4.51e-35,1.10e-04)	4.35e-22(9.47e-38,2.00e-06)	4.86e-18(1.28e-36,1.85e+01)	1.01e-20(2.68e-37,3.79e-04)	1.14e-16(0.00e+00,.)
Maximum FVC	Combined Fixed Effect	0.05(-0.01,0.11)	0.12**(0.04,0.21)	-0.19(-0.53,0.16)	-0.14(-0.48,0.19)	0.45(-0.35,1.25)
	Combined Random Effect	0.05(-0.01,0.11)	0.12**(0.03,0.21)	-0.18(-0.52,0.16)	-0.13(-0.45,0.20)	0.46(-0.38,1.30)
	Estimated variance of random effect	1.07e-23(7.42e-39,1.53e-08)	7.43e-19(1.26e-30,4.38e-07)	1.14e-19(1.06e-38,1.22e+00)	7.52e-22(3.15e-42,1.80e-01)	8.56e-20(2.12e-40,3.47e+01)
FEV1/FVC ratio	Combined Fixed Effect	0.03(-0.06,0.11)	-0.02(-0.14,0.10)	0.14(-0.32,0.60)	-0.02(-0.47,0.42)	-0.05(-1.11,1.02)
	Combined Random Effect	0.01(-0.07,0.09)	-0.02(-0.14,0.10)	0.10(-0.36,0.56)	0.01(-0.41,0.43)	0.01(-1.03,1.05)
	Estimated variance of random effect	7.19e-04(1.55e-09,3.34e+02)	2.25e-03(1.28e-07,3.96e+01)	6.94e-22(4.79e-41,1.00e-02)	4.75e-12(3.46e-30,6.51e+06)	1.19e-15(6.57e-39,2.17e+08)

Outcomes z-scored within cohorts
Cohort specific estimates are suppressed due to the small number of carriers
*p<0.05, **p<0.01, ***p<0.001, ****p<0.0001

S4 Table. Association of PI status with transformed lung function, adjusted for age and sex, in never smokers

Outcome	Cohort	Regression Coefficient (95% CI)				
		MS VS. MM	MZ VS. MM	SS VS. MM	SZ VS. MM	ZZ VS. MM
Sqrt(Maximum FVC)	Combined Fixed Effect	0.05(-0.01,0.11)	0.12**(0.04,0.21)	-0.18(-0.53,0.16)	-0.12(-0.46,0.21)	0.51(-0.29,1.30)
	Combined Random Effect	0.05(-0.01,0.11)	0.12**(0.03,0.20)	-0.20(-0.53,0.14)	-0.10(-0.42,0.22)	0.52(-0.31,1.35)
	Estimated variance of random effect	1.07e-23(1.46e-38,7.87e-09)	6.42e-19(8.06e-35,5.12e-03)	1.70e-20(3.41e-38,8.53e-03)	3.74e-21(4.78e-36,2.92e-06)	9.35e-18(5.78e-38,1.51e+03)
$(FEV1/FVC\ ratio)^3$	Combined Fixed Effect	0.01(-0.08,0.09)	-0.02(-0.14,0.10)	0.09(-0.38,0.56)	-0.09(-0.55,0.36)	-0.17(-1.26,0.92)
	Combined Random Effect	-0.00(-0.09,0.08)	-0.03(-0.16,0.10)	0.05(-0.42,0.53)	-0.13(-0.63,0.37)	-0.14(-1.21,0.93)
	Estimated variance of random effect	7.63e-04(7.09e-10,8.22e+02)	3.46e-03(2.47e-06,4.85e+00)	7.76e-17(6.18e-35,9.74e+01)	5.94e-02(1.54e-04,2.28e+01)	5.75e-17(3.92e-36,8.43e+02)

Outcomes z-scored within cohorts
Cohort specific estimates are suppressed due to the small number of carriers
*p<0.05, **p<0.01, ***p<0.001, ****p<0.0001

S5 Table. Association of PI status with lung function, adjusted for age and sex, in COPD cases

		Regression Coefficient (95% CI)	
Outcome	Cohort	MS VS. MM	MZ VS. MM
Maximum FEV1	Combined Fixed Effect	0.06(-0.05,0.18)	0.01(-0.15,0.18)
	Combined Random Effect	0.04(-0.07,0.15)	-0.00(-0.17,0.16)
	Estimated variance of random effect	5.22e-20(1.89e-33,1.44e-06)	1.87e-20(2.05e-37,1.71e-03)
Maximum FVC	Combined Fixed Effect	-0.04(-0.18,0.09)	0.12(-0.08,0.32)
	Combined Random Effect	-0.05(-0.18,0.08)	0.10(-0.10,0.29)
	Estimated variance of random effect	4.09e-18(5.45e-32,3.07e-04)	3.97e-15(2.48e-30,6.34e+00)
FEV1/FVC ratio	Combined Fixed Effect	0.21*(0.01,0.41)	-0.10(-0.41,0.20)
	Combined Random Effect	0.20*(0.01,0.38)	-0.06(-0.35,0.24)
	Estimated variance of random effect	1.47e-18(0.00e+00,.)	4.19e-23(2.04e-37,8.62e-09)

Outcomes z-scored within cohorts
Cohort specific estimates are suppressed due to the small number of carriers
Meta-analyses for PI-SS, SZ and ZZ were not possible as there were fewer than three cohorts with adequate data
p<0.05, **p<0.01, ***p<0.001, ****p<0.0001

S6 Table. Association of PI status with lung function adjusted for age, sex, height and height-squared

Outcome	Cohort	Regression Coefficient (95% CI)				
		MS VS. MM	MZ VS. MM	SS VS. MM	SZ VS. MM	ZZ VS. MM
Maximum FEV1	BO	-0.16(-0.46,0.14)	0.17(-0.40,0.74)	[a]		
	CaPS	0.05(-0.11,0.21)	0.09(-0.12,0.30)			
	ELSA	0.05(-0.02,0.12)	0.06(-0.05,0.16)			
	HAS	-0.41(-0.87,0.05)	0.11(-0.34,0.56)			
	HCS	0.00(-0.09,0.09)	-0.01(-0.15,0.12)			
	LBC1921	-0.08(-0.31,0.16)	0.09(-0.27,0.44)			
	NSHD	-0.02(-0.12,0.08)	0.08(-0.08,0.23)			
	WHII	0.04(-0.03,0.10)	0.11(-0.00,0.21)			
	Combined Fixed Effect	0.02(-0.02,0.06)	0.07*(0.01,0.12)	0.03(-0.20,0.25)	0.02(-0.21,0.24)	-0.31(-0.87,0.26)
	Combined Random Effect	0.02(-0.02,0.06)	0.07*(0.01,0.12)	0.03(-0.20,0.25)	0.03(-0.20,0.26)	-0.35(-0.91,0.20)
	Estimated variance of random effect	1.32e-13(1.51e-27,1.15e+01)	1.03e-17(1.80e-32,5.87e-03)	4.91e-14(7.47e-31,3.22e+03)	1.59e-23(5.06e-38,5.00e-09)	1.53e-06(8.51e-32,2.74e+19)
Maximum FVC	BO	-0.11(-0.37,0.15)	0.06(-0.43,0.56)	[a]		
	CaPS	0.07(-0.09,0.23)	0.13(-0.08,0.34)			
	ELSA	0.03(-0.04,0.09)	0.04(-0.06,0.14)			
	HAS	-0.30(-0.69,0.09)	0.35(-0.04,0.74)			
	HCS	-0.01(-0.08,0.07)	0.07(-0.05,0.18)			
	LBC1921	-0.09(-0.31,0.14)	0.10(-0.23,0.44)			
	NSHD	-0.02(-0.12,0.07)	0.08(-0.06,0.23)			
	WHII	-0.02(-0.08,0.05)	0.10(-0.00,0.20)			
	Combined Fixed Effect	-0.00(-0.04,0.03)	0.08**(0.03,0.13)	-0.11(-0.32,0.11)	0.02(-0.20,0.23)	-0.16(-0.69,0.36)
	Combined Random Effect	-0.01(-0.04,0.03)	0.08**(0.03,0.13)	-0.10(-0.30,0.11)	0.01(-0.20,0.22)	-0.19(-0.71,0.34)
	Estimated variance of random effect	6.12e-14(3.19e-29,1.17e+02)	1.25e-13(5.20e-27,3.01e+00)	9.71e-17(8.57e-33,1.10e+00)	8.32e-19(3.11e-33,2.22e-04)	1.31e-14(4.02e-33,4.26e+04)

FEV1/FVC ratio	BO	-0.28(-0.70,0.14)	0.26(-0.55,1.07)	[a]		
	CaPS	0.01(-0.16,0.18)	0.02(-0.20,0.25)			
	ELSA	0.04(-0.06,0.14)	0.02(-0.12,0.17)			
	HAS	-0.20(-0.80,0.40)	-0.28(-0.84,0.28)			
	HCS	0.03(-0.10,0.15)	-0.12(-0.31,0.07)			
	LBC1921	-0.01(-0.34,0.31)	-0.13(-0.63,0.36)			
	NSHD	-0.01(-0.16,0.13)	-0.06(-0.28,0.16)			
	WHII	0.11*(0.01,0.21)	0.03(-0.13,0.19)			
	Combined Fixed Effect	0.04(-0.01,0.09)	-0.02(-0.10,0.06)	0.23(-0.09,0.55)	0.02(-0.30,0.33)	-0.67(-1.46,0.11)
	Combined Random Effect	0.04(-0.01,0.09)	-0.02(-0.10,0.06)	0.23(-0.09,0.55)	0.02(-0.30,0.33)	-0.55(-1.41,0.32)
	Estimated variance of random effect	2.26e-21(9.99e-37,5.13e-06)	2.27e-17(1.70e-30,3.03e-04)	1.15e-17(1.90e-33,6.99e-02)	1.19e-19(1.19e-37,1.19e-01)	1.23e-01(5.10e-05,2.97e+02)

Outcomes z-scored within cohorts
*p<0.05, **p<0.01, ***p<0.001, ****p<0.0001
[a] Cohort specific estimates are suppressed for PI-SS, SZ and ZZ due to the small number of carriers

S7 Table. Association of PI status with lung function adjusted for age, sex, height and height-squared, outcomes transformed

Outcome	Cohort	Regression Coefficient (95% CI)				
		MS VS. MM	MZ VS. MM	SS VS. MM	SZ VS. MM	ZZ VS. MM
Sqrt(Maximum FVC)	BO	-0.15(-0.42,0.11)	0.07(-0.44,0.58)	b		
	CaPS	0.06(-0.09,0.22)	0.11(-0.10,0.32)			
	ELSA	0.02(-0.04,0.09)	0.03(-0.07,0.13)			
	HAS	-0.29(-0.68,0.10)	0.33(-0.06,0.72)			
	HCS	-0.00(-0.08,0.07)	0.06(-0.06,0.17)			
	LBC1921	-0.10(-0.33,0.13)	0.10(-0.25,0.44)			
	NSHD	-0.02(-0.11,0.08)	0.09(-0.06,0.23)			
	WHII	-0.02(-0.08,0.04)	0.09(-0.00,0.19)			
	Combined Fixed Effect	-0.00(-0.04,0.03)	0.07**(0.02,0.12)	-0.09(-0.31,0.12)	0.02(-0.19,0.23)	-0.17(-0.69,0.36)
	Combined Random Effect	-0.01(-0.04,0.03)	0.07**(0.02,0.12)	-0.09(-0.29,0.12)	0.02(-0.19,0.23)	-0.20(-0.71,0.32)
	Estimated variance of random effect	2.16e-20(3.13e-36,1.49e-04)	6.57e-18(0.00e+00,.)	8.37e-16(2.36e-30,2.97e-01)	3.40e-17(0.00e+00,.)	1.51e-16(6.00e-34,3.79e+01)
$(FEV1/FVC\ ratio)^3$	BO	-0.25(-0.68,0.18)	0.21(-0.62,1.04)	b		
	CaPS	-0.01(-0.19,0.16)	0.01(-0.22,0.25)			
	ELSA	0.04(-0.06,0.13)	0.04(-0.10,0.19)			
	HAS	-0.11(-0.69,0.47)	-0.37(-0.93,0.20)			
	HCS	0.01(-0.11,0.14)	-0.06(-0.25,0.12)			
	LBC1921	-0.06(-0.38,0.27)	-0.12(-0.61,0.37)			
	NSHD	-0.02(-0.16,0.13)	-0.10(-0.32,0.12)			
	WHII	0.10*(0.00,0.21)	0.05(-0.11,0.21)			
	Combined Fixed Effect	0.03(-0.02,0.08)	-0.01(-0.08,0.07)	0.19(-0.13,0.50)	-0.06(-0.37,0.26)	-0.60(-1.38,0.19)

	Combined Random Effect	0.03(-0.02,0.08)	-0.01(-0.08,0.07)	0.18(-0.13,0.50)	NOCONVERGENCE[a]	-0.56(-1.33,0.22)
	Estimated variance of random effect	2.70e-21(3.30e-35,2.22e-07)	1.26e-17(7.38e-31,2.15e-04)	1.82e-19(1.20e-35,2.78e-03)	NOCONVERGENCE[a]	5.89e-12(9.75e-32,3.56e+08)

Outcomes z-scored within cohorts
*p<0.05, **p<0.01, ***p<0.001, ****p<0.0001
[a] Random effects model failed to converge
[b] Cohort specific estimates are suppressed for PI-SS, SZ and ZZ due to the small number of carriers

S8 Table. Association of PI status with lung function, adjusted for age, sex, height, height-squared and smoking status in COPD cases

		Regression Coefficient (95% CI)	
Outcome	Cohort	MS VS. MM	MZ VS. MM
Maximum FEV1	Combined Fixed Effect	0.06(-0.05,0.17)	-0.02(-0.18,0.14)
	Combined Random Effect	0.04(-0.07,0.14)	-0.05(-0.20,0.11)
	Estimated variance of random effect	1.86e-20(4.01e-36,8.62e-05)	1.06e-18(1.27e-32,8.95e-05)
Maximum FVC	Combined Fixed Effect	-0.06(-0.19,0.06)	0.06(-0.13,0.25)
	Combined Random Effect	-0.06(-0.18,0.06)	0.04(-0.14,0.21)
	Estimated variance of random effect	3.06e-24(1.30e-36,7.19e-12)	6.24e-21(2.66e-37,1.47e-04)
FEV1/FVC ratio	Combined Fixed Effect	0.23*(0.02,0.43)	-0.09(-0.40,0.21)
	Combined Random Effect	0.22*(0.04,0.41)	-0.05(-0.35,0.24)
	Estimated variance of random effect	1.63e-21(6.35e-35,4.20e-08)	1.25e-18(4.60e-33,3.42e-04)

Outcomes z-scored within cohorts
Cohort specific estimates are suppressed due to the small number of carriers
Meta-analyses for PI-SS, SZ and ZZ were not possible as there were fewer than three cohorts with adequate data*p<0.05, **p<0.01, ***p<0.001, ****p<0.0001

S20 Table. Association of PI status with lung function, adjusted for age and sex, in COPD cases with outcomes transformed

		Regression Coefficient (95% CI)	
Outcome	Cohort	MS VS. MM	MZ VS. MM
Sqrt(Maximum FVC)	Combined Fixed Effect	-0.05(-0.19,0.09)	0.12(-0.09,0.33)
	Combined Random Effect	-0.05(-0.19,0.08)	0.10(-0.11,0.30)
	Estimated variance of random effect	1.50e-20(1.42e-35,1.59e-05)	6.87e-13(1.79e-31,2.64e+06)
$(FEV1/FVC\ ratio)^3$	Combined Fixed Effect	0.12*(0.02,0.22)	-0.05(-0.19,0.10)
	Combined Random Effect	0.11*(0.02,0.20)	0.00(-0.14,0.14)
	Estimated variance of random effect	1.24e-20(9.41e-49,1.63e+08)	4.31e-19(9.86e-38,1.88e+00)

Outcomes z-scored within cohorts
Cohort specific estimates are suppressed due to the small number of carriers
Meta-analyses for PI-SS, SZ and ZZ were not possible as there were fewer than three cohorts with adequate data
*p<0.05, **p<0.01, ***p<0.001, ****p<0.0001

S21 Table. Association of PI-MZ with lung function with +/-3SDs removed from outcome before standardisation, adjusted for age, sex, height and height-squared

Outcome	Cohort	Regression Coefficient (95% CI)
Maximum FEV1	Combined Fixed Effect	0.07*(0.01,0.13)
	Combined Random Effect	0.07*(0.01,0.13)
	Estimated variance of random effect	1.48e-18(0.00e+00,.)
Maximum FVC	Combined Fixed Effect	0.08**(0.03,0.13)
	Combined Random Effect	0.08**(0.03,0.13)
	Estimated variance of random effect	1.06e-12(1.14e-25,9.94e+00)
Outcomes z-scored within cohorts<br>*p<0.05, **p<0.01, ***p<0.001, ****p<0.0001		

S9 Table. Association of PI status with lung function adjusted for age, sex, height, height-squared and height-cubed

Outcome	Cohort	Regression Coefficient (95% CI)				
		MS VS. MM	MZ VS. MM	SS VS. MM	SZ VS. MM	ZZ VS. MM
Maximum FEV1	BO	-0.18(-0.48,0.12)	0.16(-0.41,0.73)	[a]		
	CaPS	0.05(-0.11,0.21)	0.09(-0.12,0.30)			
	ELSA	0.05(-0.02,0.12)	0.06(-0.04,0.17)			
	HAS	-0.41(-0.87,0.05)	0.10(-0.35,0.56)			
	HCS	0.00(-0.09,0.09)	-0.01(-0.15,0.12)			
	LBC1921	-0.08(-0.31,0.16)	0.08(-0.27,0.44)			
	NSHD	-0.02(-0.12,0.08)	0.08(-0.08,0.23)			
	WHII	0.04(-0.03,0.10)	0.11(-0.00,0.21)			
	Combined Fixed Effect	0.02(-0.02,0.06)	0.07*(0.01,0.12)	0.02(-0.20,0.25)	0.02(-0.21,0.25)	-0.30(-0.87,0.26)
	Combined Random Effect	0.02(-0.02,0.06)	0.07*(0.01,0.12)	0.03(-0.20,0.25)	0.03(-0.20,0.26)	-0.35(-0.91,0.21)
	Estimated variance of random effect	9.32e-18(7.18e-30,1.21e-05)	1.66e-19(6.53e-35,4.20e-04)	2.85e-15(4.20e-32,1.93e+02)	8.24e-16(4.26e-30,1.59e-01)	3.10e-12(4.97e-33,1.93e+09)
Maximum FVC	BO	-0.12(-0.38,0.14)	0.07(-0.43,0.57)	[a]		
	CaPS	0.07(-0.09,0.23)	0.13(-0.08,0.34)			
	ELSA	0.03(-0.04,0.09)	0.04(-0.06,0.14)			
	HAS	-0.30(-0.69,0.09)	0.34(-0.06,0.73)			
	HCS	-0.01(-0.08,0.07)	0.07(-0.05,0.18)			
	LBC1921	-0.09(-0.31,0.14)	0.11(-0.23,0.44)			
	NSHD	-0.02(-0.12,0.07)	0.08(-0.06,0.23)			
	WHII	-0.02(-0.08,0.05)	0.10(-0.00,0.20)			
	Combined Fixed Effect	-0.00(-0.04,0.03)	0.08**(0.03,0.14)	-0.11(-0.32,0.11)	0.02(-0.19,0.23)	-0.16(-0.69,0.36)
	Combined Random Effect	-0.01(-0.04,0.03)	0.08**(0.03,0.13)	-0.10(-0.30,0.11)	0.02(-0.19,0.23)	-0.18(-0.71,0.34)
	Estimated variance of random effect	4.40e-16(9.54e-32,2.02e+00)	7.69e-14(4.31e-27,1.37e+00)	4.85e-15(3.28e-31,7.17e+01)	1.66e-13(7.47e-30,3.68e+03)	7.50e-16(2.11e-35,2.67e+04)

FEV1/FVC ratio	BO	-0.31(-0.74,0.11)	0.23(-0.58,1.04)	[a]		
	CaPS	0.01(-0.16,0.18)	0.02(-0.20,0.25)			
	ELSA	0.04(-0.06,0.14)	0.02(-0.12,0.17)			
	HAS	-0.19(-0.79,0.41)	-0.27(-0.83,0.30)			
	HCS	0.03(-0.10,0.15)	-0.12(-0.31,0.07)			
	LBC1921	-0.01(-0.34,0.31)	-0.14(-0.64,0.36)			
	NSHD	-0.01(-0.16,0.13)	-0.06(-0.28,0.16)			
	WHII	0.11*(0.01,0.21)	0.03(-0.13,0.19)			
	Combined Fixed Effect	0.04(-0.01,0.09)	-0.02(-0.10,0.06)	0.23(-0.08,0.55)	0.02(-0.30,0.33)	-0.67(-1.46,0.11)
	Combined Random Effect	0.04(-0.01,0.09)	-0.02(-0.10,0.06)	0.23(-0.09,0.55)	0.02(-0.30,0.33)	-0.55(-1.41,0.32)
	Estimated variance of random effect	5.96e-19(1.82e-32,1.95e-05)	5.70e-14(0.00e+00,.)	1.50e-16(9.10e-31,2.48e-02)	9.05e-11(1.38e-27,5.94e+06)	1.22e-01(4.76e-05,3.13e+02)

Outcomes z-scored within cohorts
*p<0.05, **p<0.01, ***p<0.001, ****p<0.0001
[a] Cohort specific estimates are suppressed for PI-SS, SZ and ZZ due to the small number of carriers

S10 Table. Association of PI status with lung function adjusted for age, sex, height, height-squared and height-cubed, outcomes transformed

Outcome	Cohort	Regression Coefficient (95% CI)				
		MS VS. MM	MZ VS. MM	SS VS. MM	SZ VS. MM	ZZ VS. MM
Sqrt(Maximum FVC)	BO	-0.16(-0.43,0.11)	0.08(-0.43,0.59)	[a]		
	CaPS	0.06(-0.09,0.22)	0.11(-0.09,0.32)			
	ELSA	0.02(-0.04,0.09)	0.03(-0.07,0.13)			
	HAS	-0.29(-0.68,0.10)	0.32(-0.07,0.71)			
	HCS	-0.00(-0.08,0.07)	0.06(-0.06,0.17)			
	LBC1921	-0.10(-0.33,0.13)	0.10(-0.24,0.44)			
	NSHD	-0.02(-0.11,0.08)	0.09(-0.05,0.23)			
	WHII	-0.02(-0.08,0.04)	0.09(-0.00,0.19)			
	Combined Fixed Effect	-0.00(-0.04,0.03)	0.07**(0.02,0.13)	-0.10(-0.31,0.11)	0.02(-0.19,0.23)	-0.16(-0.69,0.36)
	Combined Random Effect	-0.01(-0.04,0.03)	0.07**(0.02,0.12)	-0.09(-0.29,0.12)	0.02(-0.19,0.23)	-0.19(-0.71,0.32)
	Estimated variance of random effect	1.85e-19(7.62e-32,4.47e-07)	3.78e-16(4.59e-31,3.11e-01)	3.68e-15(3.95e-33,3.43e+03)	1.22e-13(5.83e-28,2.54e+01)	2.38e-15(0.00e+00,.)
$(FEV1/FVC\ ratio)^3$	BO	-0.29(-0.71,0.14)	0.17(-0.66,1.00)	[a]		
	CaPS	-0.01(-0.19,0.16)	0.01(-0.22,0.25)			
	ELSA	0.04(-0.06,0.13)	0.04(-0.10,0.19)			
	HAS	-0.10(-0.68,0.48)	-0.35(-0.92,0.22)			
	HCS	0.01(-0.11,0.14)	-0.06(-0.25,0.12)			
	LBC1921	-0.06(-0.38,0.27)	-0.13(-0.63,0.36)			
	NSHD	-0.02(-0.16,0.13)	-0.10(-0.32,0.12)			
	WHII	0.10*(0.00,0.21)	0.05(-0.11,0.21)			
	Combined Fixed Effect	0.03(-0.02,0.08)	-0.01(-0.08,0.07)	0.19(-0.13,0.50)	-0.06(-0.37,0.26)	-0.59(-1.38,0.19)
	Combined Random Effect	0.03(-0.02,0.08)	-0.01(-0.08,0.07)	0.19(-0.13,0.50)	-0.05(-0.38,0.27)	-0.55(-1.33,0.22)
	Estimated variance of random effect	1.01e-18(8.43e-33,1.20e-04)	1.64e-16(1.28e-28,2.10e-04)	1.34e-17(1.32e-35,1.36e+01)	4.00e-03(3.93e-07,4.08e+01)	5.34e-12(2.39e-31,1.19e+08)

Outcomes z-scored within cohorts
*p<0.05, **p<0.01, ***p<0.001, ****p<0.0001
[a] Cohort specific estimates are suppressed for PI-SS, SZ and ZZ due to the small number of carriers

S11 Table. Association of PI status with physical capability, adjusted for age and sex

Outcome	Cohort	Coefficient[a] (95% CI)			
		MS VS. MM	MZ VS. MM	SS VS. MM	SZ VS. MM
Grip strength	ELSA	-0.00(-0.06,0.05)	-0.02(-0.11,0.06)	[c]	
	HAS	0.02(-0.17,0.21)	0.07(-0.18,0.33)		
	HCS	0.01(-0.07,0.08)	0.12*(0.01,0.23)		
	LBC1921	-0.12(-0.33,0.08)	0.29(-0.02,0.60)		
	NSHD	0.05(-0.04,0.15)	0.13(-0.01,0.27)		
	Combined Fixed Effect	0.01(-0.03,0.05)	0.06*(0.01,0.12)	0.00(-0.23,0.23)	0.00(-0.22,0.22)
	Combined Random Effect	0.01(-0.03,0.05)	0.08(-0.00,0.16)	0.03(-0.20,0.26)	-0.01(-0.23,0.20)
	Estimated variance of random effect	3.66e-16(4.15e-37,3.23e+05)	2.98e-03(1.75e-04,5.08e-02)	8.78e-10(1.18e-27,6.53e+08)	3.66e-16(5.67e-37,2.37e+05)
Chair rise speed	ELSA	-0.04(-0.14,0.05)	-0.03(-0.17,0.11)	[c]	
	HAS	0.21(-0.35,0.78)	0.32(-0.25,0.89)		
	HCS	-0.15(-0.31,0.02)	-0.01(-0.25,0.24)		
	NSHD	-0.01(-0.14,0.13)	0.10(-0.09,0.30)		
	Combined Fixed Effect	-0.04(-0.11,0.02)	0.03(-0.07,0.13)	0.29(-0.13,0.72)	0.03(-0.35,0.40)
	Combined Random Effect	-0.05(-0.12,0.02)	0.02(-0.08,0.12)	0.27(-0.15,0.70)	-0.00(-0.77,0.77)
	Estimated variance of random effect	9.52e-16(0.00e+00,.)	1.02e-19(4.96e-43,2.08e+04)	8.75e-19(2.29e-37,3.34e+00)	3.74e-01(3.87e-02,3.61e+00)
Walk speed	ELSA	0.06(-0.05,0.17)	0.09(-0.07,0.25)	[c]	
	HAS	-0.31(-0.81,0.19)	0.52(-0.00,1.05)		
	HCS	-0.04(-0.18,0.10)	0.05(-0.15,0.25)		
	LBC1921	0.19(-0.13,0.51)	0.20(-0.26,0.67)		
	WHII	-0.07(-0.16,0.01)	0.03(-0.10,0.17)		
	Combined Fixed Effect	-0.02(-0.08,0.03)	0.07(-0.02,0.16)	0.16(-0.17,0.48)	-0.28(-0.63,0.07)
	Combined Random Effect	-0.02(-0.09,0.05)	0.07(-0.01,0.16)	0.15(-0.17,0.48)	-0.32(-0.80,0.17)
	Estimated variance of random effect	8.25e-04(9.04e-07,7.53e-01)	1.65e-17(1.70e-32,1.59e-02)	2.26e-13(4.27e-62,1.19e+36)	1.13e-01(5.11e-03,2.51e+00)

TUG speed	BO	-0.08(-0.48,0.32)	0.70(-0.07,1.48)	[c]	
	CaPS	-0.10(-0.33,0.14)	-0.01(-0.34,0.31)		
	HAS	-0.41(-0.88,0.07)	0.42(-0.09,0.93)		
	HCS	-0.05(-0.19,0.09)	0.03(-0.17,0.24)		
	NSHD	0.09(-0.07,0.25)	-0.00(-0.24,0.23)		
	Combined Fixed Effect	-0.02(-0.11,0.07)	0.06(-0.08,0.19)	0.07(-0.49,0.62)	-0.80**(-1.31,-0.28)[d]
	Combined Random Effect	-0.02(-0.12,0.07)	0.06(-0.07,0.19)	0.05(-0.51,0.61)	-0.75*(-1.32,-0.18)[d]
	Estimated variance of random effect	6.01e-17(6.36e-46,5.68e+12)	1.29e-12(1.15e-191,1.46e+167)	1.27e-10[b]	6.60e-02(1.12e-04,3.87e+01)
Inability to balance for 5s	BO	1.28(0.46,3.54)	-	[c]	
	CaPS	0.62(0.32,1.22)	1.26(0.56,2.81)		
	ELSA	1.21(0.89,1.65)	1.21(0.77,1.90)		
	HAS	2.01(0.68,5.91)	1.07(0.31,3.71)		
	HCS	1.08(0.69,1.69)	0.52(0.22,1.22)		
	NSHD	0.96(0.49,1.87)	0.93(0.33,2.58)		
	Combined Fixed Effect	1.09(0.88,1.35)	0.98(0.72,1.35)	F3CH	F3CH
	Combined Random Effect	1.09(0.88,1.35)	0.98(0.72,1.35)	F3CH	F3CH
	Estimated variance of random effect	4.33e-12(0.00e+00,.)	2.00e-08(0.00e+00,.)	F3CH	F3CH

Continuous outcomes z-scored within cohorts
Meta-analyses for PI-ZZ were not possible as there were fewer than three cohorts with adequate data
F3CH: Fewer than 3 cohorts with adequate data to perform the meta-analysis
*p<0.05, **p<0.01, ***p<0.001, ****p<0.0001
[a] Coefficients are linear regression coefficients for continuous outcomes and odds ratios for binary outcomes
[b] Standard error of the variance of the random effect not estimable
[c] Cohort specific estimates are suppressed for PI-SS and SZ due to the small number of carriers
[d] Association driven by a single observation

S12 Table. Number of COPD cases and non-cases in PI status – COPD analysis

Cohort	Number COPD cases	Number COPD non-cases	Total
BO	35	227	262
CaPS	86	1187	1273
ELSA	486	4331	4817
HAS	22	162	184
HCS	233	2278	2511
LBC1921	28	476	504
NSHD	88	1892	1980
WHII	315	3917	4232
Total	1293	14470	15763
Total (%)	8.20	91.80	100
Numbers based on individuals with a valid PI status, age, sex, height and lung function measures			

S13 Table. Association of PI status with COPD status, adjusted for age and sex

	OR for COPD[a] (95% CI)	
Cohort	MS VS. MM	MZ VS. MM
BO	2.32(0.84,6.36)	-
CaPS	1.02(0.48,2.18)	0.94(0.33,2.67)
ELSA	1.05(0.76,1.46)	1.08(0.67,1.73)
HAS	1.39(0.28,6.82)	0.66(0.08,5.43)
HCS	0.92(0.58,1.47)	1.27(0.70,2.31)
LBC1921	0.87(0.20,3.81)	1.14(0.14,9.09)
NSHD	1.40(0.75,2.64)	-
WHII	0.65(0.41,1.02)	0.99(0.54,1.81)
Combined Fixed Effect	0.97(0.79,1.18)	0.98(0.73,1.31)
Combined Random Effect	0.97(0.79,1.18)	0.98(0.73,1.31)
Estimated variance of random effect	2.23e-07(0.00e+00,.)	3.76e-17(0.00e+00,.)

Meta-analyses for PI-SS, SZ and ZZ were not possible as there were fewer than three cohorts with adequate data

*p<0.05, **p<0.01, ***p<0.001, ****p<0.0001

[a] Coefficient is odds ratio for COPD in PI class versus PI-MM

S14 Table. **Association of PI status with height (cm) adjusted for age and sex**

Cohort	Regression Coefficient (95% CI)				
	MS VS. MM	MZ VS. MM	SS VS. MM	SZ VS. MM	ZZ VS. MM
BO	2.31(-0.24,4.86)	7.33**(2.36,12.30)	[a]		
CaPS	0.27(-0.97,1.51)	0.55(-1.08,2.18)			
ELSA	0.20(-0.40,0.81)	2.02****(1.12,2.92)			
HAS	0.24(-3.04,3.51)	-0.26(-3.69,3.18)			
HCS	-0.10(-0.87,0.67)	1.23*(0.09,2.37)			
LBC1921	0.44(-1.55,2.43)	1.06(-1.89,4.00)			
NSHD	0.68(-0.11,1.47)	1.84**(0.64,3.04)			
WHII	0.23(-0.34,0.81)	1.24**(0.34,2.14)			
Combined Fixed Effect	0.28(-0.03,0.59)	1.50****(1.03,1.97)	-0.13(-2.07,1.80)	1.78(-0.13,3.69)	2.04(-2.33,6.41)
Combined Random Effect	0.28(-0.03,0.59)	1.51****(1.04,1.97)	-1.24(-5.15,2.67)	1.81(-0.10,3.71)	2.04(-2.36,6.43)
Estimated variance of random effect	2.77e-13(1.06e-26,7.27e+00)	1.67e-12(1.59e-25,1.76e+01)	1.57e+01(2.04e+00,1.20e+02)	2.22e-11(8.31e-27,5.92e+04)	1.13e-11(1.32e-29,9.74e+06)

*p<0.05, **p<0.01, ***p<0.001, ****p<0.0001

[a] Cohort specific estimates are suppressed for PI-SS, SZ and ZZ due to the small number of carriers

S15 Table. Association of PI-MZ with Height, +/-3 SDs removed from outcome, adjusted for age and sex

Cohort	Regression Coefficient (95% CI) (cm)
Combined Fixed Effect	1.53****(1.06,1.99)
Combined Random Effect	1.53****(1.06,1.99)
Estimated variance of random effect	6.08e-11(9.70e-25,3.82e+03)
*p<0.05, **p<0.01, ***p<0.001, ****p<0.0001	

S16 Table. Association of PI status with height, adjusted for age, sex, FEV1(z-score) and FVC(z-score)

Cohort	Regression Coefficient (95% CI) (cm)				
	MS VS. MM	MZ VS. MM	SS VS. MM	SZ VS. MM	ZZ VS. MM
BO	2.06(-0.28,4.40)	5.76*(1.22,10.29)	[a]		
CaPS	-0.04(-1.17,1.09)	-0.18(-1.66,1.31)			
ELSA	0.04(-0.54,0.63)	1.55***(0.69,2.40)			
HAS	0.94(-2.19,4.07)	-1.33(-4.49,1.83)			
HCS	-0.06(-0.75,0.63)	0.53(-0.50,1.55)			
LBC1921	0.75(-1.05,2.54)	0.62(-2.12,3.37)			
NSHD	0.61(-0.20,1.42)	1.20(-0.01,2.42)			
WHII	0.15(-0.43,0.72)	0.76(-0.14,1.66)			
Combined Fixed Effect	0.18(-0.12,0.48)	0.92****(0.47,1.37)	0.13(-1.69,1.95)	0.89(-0.93,2.71)	1.32(-3.19,5.84)
Combined Random Effect	0.19(-0.11,0.49)	0.91****(0.46,1.35)	-0.46(-3.30,2.37)	0.92(-0.91,2.74)	1.28(-3.24,5.79)
Estimated variance of random effect	1.25e-12(9.57e-31,1.64e+06)	2.70e-08(5.75e-29,1.27e+13)	6.31e+00(3.26e-01,1.22e+02)	5.25e-11(1.34e-25,2.05e+04)	3.66e-11(5.11e-31,2.62e+09)

*p<0.05, **p<0.01, ***p<0.001, ****p<0.0001

[a] Cohort specific estimates are suppressed for PI-SS, SZ and ZZ due to the small number of carriers

S30 Table. Association of PI-MZ with Height, +/-3 SDs removed from outcome, adjusted for age, sex, FEV1 (z-score) and FVC (z-score)

Cohort	Regression Coefficient (95% CI) (cm)
Combined Fixed Effect	0.96****(0.51,1.41)
Combined Random Effect	0.94****(0.49,1.38)
Estimated variance of random effect	3.42e-07(2.24e-107,5.21e+93)
*p<0.05, **p<0.01, ***p<0.001, ****p<0.0001	

S31 Table. Association of PI status with weight, adjusted for age and sex

Cohort	Regression Coefficient (95% CI) (kg)				
	MS VS. MM	MZ VS. MM	SS VS. MM	SZ VS. MM	ZZ VS. MM
BO	0.70(-4.83,6.22)	4.25(-6.21,14.72)	[a]		
CaPS	0.27(-2.05,2.59)	0.27(-2.78,3.31)			
ELSA	0.08(-1.21,1.38)	1.05(-0.89,2.99)			
HAS	-0.38(-7.76,7.00)	-6.10(-13.96,1.77)			
HCS	-0.36(-1.98,1.26)	0.84(-1.57,3.26)			
LBC1921	-2.88(-6.48,0.73)	3.20(-2.08,8.48)			
NSHD	0.13(-1.59,1.85)	3.26*(0.61,5.91)			
WHII	0.38(-0.80,1.56)	0.28(-1.58,2.13)			
Combined Fixed Effect	0.06(-0.60,0.71)	1.02*(0.04,2.01)	-2.73(-6.79,1.32)	1.97(-2.08,6.03)	-2.43(-11.60,6.74)
Combined Random Effect	0.03(-0.62,0.68)	1.01*(0.03,1.99)	-3.15(-7.19,0.88)	2.36(-2.41,7.14)	-2.10(-11.21,7.00)
Estimated variance of random effect	5.33e-13(6.25e-25,4.54e-01)	9.93e-15(2.68e-27,3.68e-02)	7.92e-08(1.05e-22,5.97e+07)	7.88e+00(8.09e-02,7.68e+02)	1.85e-07(4.06e-176,8.46e+161)

*p<0.05, **p<0.01, ***p<0.001, ****p<0.0001
[a] Cohort specific estimates are suppressed for PI-SS, SZ and ZZ due to the small number of carriers

S17 Table. Association of PI-MZ with Weight, +/-3 SDs removed from outcome, adjusted for age and sex

Cohort	Regression Coefficient (95% CI) (kg)
Combined Fixed Effect	0.92(-0.02,1.86)
Combined Random Effect	0.95*(0.01,1.88)
Estimated variance of random effect	2.80e-11(4.20e-24,1.86e+02)
*p<0.05, **p<0.01, ***p<0.001, ****p<0.0001	

S18 Table. Association of PI status with ln(weight), adjusted for age and sex

Cohort	Regression Coefficient (95% CI)				
	MS VS. MM	MZ VS. MM	SS VS. MM	SZ VS. MM	ZZ VS. MM
BO	-0.00(-0.07,0.07)	0.06(-0.08,0.20)	[a]		
CaPS	0.00(-0.03,0.03)	0.01(-0.03,0.05)			
ELSA	0.00(-0.02,0.02)	0.02(-0.01,0.04)			
HAS	-0.01(-0.11,0.09)	-0.07(-0.17,0.04)			
HCS	-0.00(-0.02,0.02)	0.01(-0.02,0.04)			
LBC1921	-0.04(-0.09,0.01)	0.05(-0.03,0.12)			
NSHD	0.00(-0.02,0.02)	0.04*(0.00,0.07)			
WHII	0.00(-0.01,0.02)	0.00(-0.02,0.03)			
Combined Fixed Effect	0.00(-0.01,0.01)	0.01*(0.00,0.03)	-0.03(-0.09,0.02)	0.03(-0.02,0.08)	-0.02(-0.14,0.10)
Combined Random Effect	0.00(-0.01,0.01)	0.01*(0.00,0.03)	-0.04(-0.09,0.02)	0.03(-0.03,0.09)	-0.02(-0.13,0.10)
Estimated variance of random effect	1.73e-19(3.95e-35,7.59e-04)	1.72e-19(6.57e-32,4.50e-07)	8.89e-05(2.82e-14,2.80e+05)	7.46e-04(5.62e-07,9.89e-01)	2.83e-18(1.88e-41,4.27e+05)

Outcome is ln(weight in kg)
*p<0.05, **p<0.01, ***p<0.001, ****p<0.0001
[a] Cohort specific estimates are suppressed for PI-SS, SZ and ZZ due to the small number of carriers

S19 Table. Association of PI status with BMI, adjusted for age and sex

Cohort	Regression Coefficient (95% CI) (kg/m^2)				
	MS VS. MM	MZ VS. MM	SS VS. MM	SZ VS. MM	ZZ VS. MM
BO	-0.66(-2.54,1.22)	-0.88(-4.50,2.73)	[a]		
CaPS	0.05(-0.65,0.76)	0.02(-0.90,0.95)			
ELSA	0.01(-0.44,0.46)	-0.26(-0.94,0.41)			
HAS	-1.74(-4.28,0.79)	-1.83(-4.47,0.81)			
HCS	-0.09(-0.63,0.46)	-0.05(-0.86,0.76)			
LBC1921	-1.25(-2.59,0.09)	0.93(-1.04,2.90)			
NSHD	-0.20(-0.79,0.39)	0.51(-0.40,1.42)			
WHII	0.04(-0.34,0.43)	-0.27(-0.87,0.34)			
Combined Fixed Effect	-0.08(-0.30,0.14)	-0.09(-0.43,0.24)	-0.94(-2.30,0.43)	0.20(-1.16,1.56)	-1.56(-4.65,1.52)
Combined Random Effect	-0.08(-0.30,0.14)	-0.09(-0.42,0.24)	-0.92(-2.29,0.45)	0.32(-1.15,1.80)	-1.48(-4.47,1.52)
Estimated variance of random effect	5.69e-18(1.13e-32,2.88e-03)	1.35e-18(2.10e-34,8.67e-03)	4.65e-14(3.71e-29,5.82e+01)	4.04e-01(8.81e-05,1.85e+03)	2.55e-10(2.47e-28,2.63e+08)

*p<0.05, **p<0.01, ***p<0.001, ****p<0.0001

[a] Cohort specific estimates are suppressed for PI-SS, SZ and ZZ due to the small number of carriers

S20 Table. Association of PI status with ln(BMI), adjusted for age and sex

Cohort	Regression Coefficient (95% CI)				
	MS VS. MM	MZ VS. MM	SS VS. MM	SZ VS. MM	ZZ VS. MM
BO	-0.03(-0.10,0.04)	-0.03(-0.16,0.10)	[a]		
CaPS	0.00(-0.02,0.03)	0.00(-0.03,0.04)			
ELSA	0.00(-0.02,0.02)	-0.01(-0.03,0.02)			
HAS	-0.07(-0.16,0.02)	-0.06(-0.15,0.03)			
HCS	-0.00(-0.02,0.02)	-0.00(-0.03,0.03)			
LBC1921	-0.05(-0.10,0.00)	0.04(-0.04,0.11)			
NSHD	-0.01(-0.03,0.01)	0.02(-0.02,0.05)			
WHII	0.00(-0.01,0.02)	-0.01(-0.03,0.01)			
Combined Fixed Effect	-0.00(-0.01,0.00)	-0.00(-0.02,0.01)	-0.03(-0.08,0.02)	0.01(-0.04,0.06)	-0.05(-0.16,0.06)
Combined Random Effect	-0.00(-0.01,0.00)	-0.00(-0.01,0.01)	-0.03(-0.08,0.02)	0.01(-0.04,0.06)	-0.05(-0.15,0.06)
Estimated variance of random effect	1.09e-20(0.00e+00,.)	2.48e-16(2.02e-28,3.03e-04)	1.62e-17(2.73e-33,9.64e-02)	2.41e-04(1.27e-11,4.57e+03)	1.95e-18(1.08e-35,3.54e-01)

Outcome is ln(BMI in kg/m^2)
*p<0.05, **p<0.01, ***p<0.001, ****p<0.0001
[a] Cohort specific estimates are suppressed for PI-SS, SZ and ZZ due to the small number of carriers

Cystic Fibrosis

S21 Table. Cystic fibrosis deltaF508 carrier frequency

Cohort	Number of carriers[a]	Number of non-carriers[a]	Total, N[a]	Minor Allele Frequency	% female	Mean age (SE)
BO	5	258	263	0.010	54.75	69.60(0.26)
CaPS	39	1254	1293	0.015	0	53.80(0.14)
ELSA	170	4931	5101	0.017	53.85	65.62(0.13)
HAS	8	184	192	0.021	34.9	76.36(0.16)
HCS	70	2495	2565	0.014	47.02	66.09(0.06)
LBC1921	12	506	518	0.012	58.88	79.06(0.03)
NSHD	49	1912	1961	0.012	52.07	53.45(0.00)
WHII	99	3314	3413	0.015	25.99	60.63(0.10)
Combined	452	14854	15306	0.015	41.66	62.69(0.07)

[a] Total number of individuals for each mutation is total number with a valid genotype, sex, age and lung function

S22 Table. Association of deltaF508 carrier status with lung function

Outcome	Cohort	Regression Coefficient (95% CI) (age and sex adjusted model)	Regression Coefficient (95% CI) (current smokers, age and sex adjusted model)	Regression Coefficient (95% CI) (ex smokers, age and sex adjusted model)	Regression Coefficient (95% CI) (never smokers, age and sex adjusted model)	Regression Coefficient (95% CI) (COPD cases, age and sex adjusted model)	Regression Coefficient (95% CI) (age, sex, height and height-squared adjusted model)
Maximum FEV1	BO	-0.33(-1.00,0.33)	[a]				-0.23(-0.86,0.39)
	CaPS	0.05(-0.23,0.33)					0.01(-0.26,0.27)
	ELSA	-0.02(-0.14,0.09)					-0.02(-0.13,0.09)
	HAS	-0.14(-0.73,0.46)					-0.19(-0.76,0.37)
	HCS	0.05(-0.13,0.23)					-0.01(-0.18,0.16)
	LBC1921	0.01(-0.44,0.45)					0.13(-0.29,0.54)
	NSHD	-0.04(-0.26,0.17)					-0.10(-0.29,0.10)
	WHII	-0.05(-0.20,0.09)					-0.05(-0.18,0.09)
	Combined Fixed Effect	-0.02(-0.09,0.05)	-0.05(-0.23,0.12)	0.02(-0.08,0.12)	-0.05(-0.16,0.07)	0.09(-0.10,0.27)	-0.03(-0.10,0.03)
	Combined Random Effect	-0.02(-0.09,0.05)	-0.06(-0.23,0.12)	0.02(-0.08,0.12)	-0.04(-0.16,0.07)	0.09(-0.08,0.27)	-0.03(-0.10,0.03)
	Estimated variance of random effect	4.07e-14(2.19e-26,7.56e-02)	5.10e-18(0.00e+00,.)	7.32e-17(5.89e-32,9.10e-02)	1.41e-22(3.51e-40,5.64e-05)	5.38e-18(1.71e-35,1.69e+00)	3.81e-17(5.16e-33,2.81e-01)
Maximum FVC	BO	-0.17(-0.79,0.45)	[a]				-0.04(-0.59,0.52)
	CaPS	-0.16(-0.45,0.14)					-0.21(-0.48,0.05)
	ELSA	-0.09(-0.20,0.02)					-0.11(-0.21,0.00)
	HAS	0.05(-0.47,0.57)					-0.01(-0.49,0.48)
	HCS	0.10(-0.06,0.26)					0.01(-0.13,0.16)
	LBC1921	-0.17(-0.61,0.27)					-0.03(-0.43,0.36)
	NSHD	0.01(-0.20,0.21)					-0.05(-0.23,0.13)
	WHII	-0.09(-0.23,0.05)					-0.08(-0.20,0.04)
	Combined Fixed Effect	-0.06(-0.12,0.01)	-0.12(-0.29,0.05)	-0.00(-0.10,0.09)	-0.08(-0.20,0.03)	-0.07(-0.29,0.16)	-0.08*(-0.14,-0.02)
	Combined Random Effect	-0.05(-0.12,0.02)	-0.12(-0.29,0.05)	0.00(-0.10,0.10)	-0.08(-0.19,0.03)	-0.08(-0.29,0.14)	-0.07*(-0.13,-0.01)

	Estimated variance of random effect	5.60e-08(7.09e-24,4.42e+08)	6.12e-15(6.97e-30,5.37e+00)	6.43e-15(7.91e-27,5.23e-03)	1.23e-22(6.83e-39,2.23e-06)	3.15e-23(4.54e-42,2.18e-04)	1.93e-17(2.22e-29,1.67e-05)
FEV1/FVC ratio	BO	-0.39(-1.27,0.49)	[a]				-0.43(-1.31,0.45)
	CaPS	0.42**(0.14,0.69)					0.42**(0.14,0.70)
	ELSA	0.08(-0.07,0.24)					0.11(-0.05,0.26)
	HAS	-0.44(-1.16,0.28)					-0.44(-1.16,0.28)
	HCS	-0.05(-0.28,0.19)					0.01(-0.23,0.24)
	LBC1921	0.35(-0.22,0.92)					0.33(-0.24,0.91)
	NSHD	-0.11(-0.39,0.17)					-0.08(-0.36,0.20)
	WHII	0.04(-0.16,0.23)					0.03(-0.17,0.22)
	Combined Fixed Effect	0.05(-0.04,0.15)	0.10(-0.13,0.34)	0.05(-0.08,0.18)	0.04(-0.11,0.19)	0.26(-0.07,0.59)	0.07(-0.02,0.16)
	Combined Random Effect	0.06(-0.05,0.17)	0.08(-0.24,0.39)	0.07(-0.06,0.19)	0.06(-0.09,0.20)	0.23(-0.09,0.55)	0.08(-0.01,0.17)
	Estimated variance of random effect	4.58e-03(1.25e-06,1.67e+01)	5.84e-02(3.72e-03,9.16e-01)	1.22e-18(3.66e-34,4.06e-03)	1.82e-07(2.94e-22,1.12e+08)	2.20e-17(5.96e-34,8.15e-01)	1.01e-11(1.73e-26,5.88e+03)

Outcomes z-scored within cohorts
*p<0.05, **p<0.01, ***p<0.001, ****p<0.0001
[a] Cohort specific estimates are suppressed for smoking and COPD strata due to the small number of carriers

S23 Table. Association of deltaF508 carrier status with lung function, outcomes transformed

Outcome	Cohort	Regression Coefficient (95% CI) (age and sex adjusted model)	Regression Coefficient (95% CI) (current smokers, age and sex adjusted model)	Regression Coefficient (95% CI) (ex smokers, age and sex adjusted model)	Regression Coefficient (95% CI) (never smokers, age and sex adjusted model)	Regression Coefficient (95% CI) (COPD cases, age and sex adjusted model)	Regression Coefficient (95% CI) (age, sex, height and height-squared adjusted model)
Sqrt(Maximum FVC)	BO	-0.16(-0.78,0.46)	[a]				-0.03(-0.60,0.53)
	CaPS	-0.14(-0.43,0.14)					-0.20(-0.46,0.06)
	ELSA	-0.08(-0.19,0.03)					-0.10(-0.20,0.01)
	HAS	0.07(-0.44,0.58)					0.01(-0.47,0.49)
	HCS	0.09(-0.07,0.25)					0.00(-0.14,0.15)
	LBC1921	-0.17(-0.61,0.28)					-0.04(-0.44,0.36)
	NSHD	0.01(-0.20,0.22)					-0.05(-0.24,0.13)
	WHII	-0.09(-0.23,0.05)					-0.08(-0.20,0.04)
	Combined Fixed Effect	-0.05(-0.12,0.02)	-0.11(-0.28,0.06)	-0.00(-0.10,0.10)	-0.09(-0.20,0.02)	-0.07(-0.31,0.17)	-0.08*(-0.14,-0.01)
	Combined Random Effect	-0.05(-0.11,0.02)	-0.11(-0.28,0.05)	0.01(-0.09,0.10)	-0.09(-0.20,0.02)	-0.08(-0.31,0.14)	-0.07*(-0.13,-0.01)
	Estimated variance of random effect	8.13e-14(6.22e-28,1.06e+01)	1.44e-10(0.00e+00,.)	1.21e-13(1.17e-26,1.25e+00)	1.35e-20(6.45e-35,2.83e-06)	2.71e-22(6.22e-37,1.18e-07)	1.63e-22(2.46e-36,1.08e-08)
$(FEV1/FVC\ ratio)^3$	BO	-0.16(-1.05,0.73)	[a]				-0.21(-1.10,0.69)
	CaPS	0.47**(0.19,0.76)					0.48**(0.19,0.77)
	ELSA	0.09(-0.06,0.24)					0.12(-0.04,0.27)
	HAS	-0.47(-1.17,0.23)					-0.48(-1.17,0.22)
	HCS	-0.02(-0.25,0.22)					0.04(-0.19,0.27)
	LBC1921	0.38(-0.20,0.95)					0.36(-0.22,0.93)
	NSHD	-0.06(-0.34,0.22)					-0.03(-0.31,0.25)
	WHII	0.01(-0.19,0.20)					0.00(-0.20,0.20)

	Combined Fixed Effect	0.07(-0.03,0.16)	0.15(-0.07,0.38)	0.06(-0.07,0.19)	0.04(-0.12,0.19)	0.13(-0.03,0.29)	0.09(-0.01,0.18)
	Combined Random Effect	0.07(-0.04,0.19)	0.09(-0.24,0.41)	0.07(-0.06,0.20)	0.06(-0.13,0.25)	0.12(-0.03,0.27)	0.09(-0.02,0.20)
	Estimated variance of random effect	7.20e-03(2.64e-05,1.96e+00)	7.61e-02(7.02e-03,8.24e-01)	7.30e-11(7.66e-27,6.95e+05)	1.47e-02(2.22e-04,9.76e-01)	6.50e-22[b]	3.68e-03(2.25e-07,6.04e+01)

Outcomes z-scored within cohorts
*p<0.05, **p<0.01, ***p<0.001, ****p<0.0001
[a] Cohort specific estimates are suppressed for smoking and COPD strata due to the small number of carriers
[b] Standard error of the variance of the random effect not estimable

S24 Table. Number of COPD cases and non-cases in deltaF508 carrier – COPD analysis

Cohort	Number COPD cases	Number COPD non-cases	Total
BO	35	228	263
CaPS	87	1189	1276
ELSA	504	4461	4965
HAS	22	168	190
HCS	240	2323	2563
LBC1921	30	488	518
NSHD	89	1867	1956
WHII	246	3162	3408
Total	1253	13886	15139
Total (%)	8.28	91.72	100
Numbers based on individuals with a valid deltaF508 genotype, age, sex, height and lung function measures			

S40 Table. Association of deltaF508 carrier status with COPD status, adjusted for age and sex

Cohort	OR for COPD[a] (95% CI)
BO	1.55(0.17,14.53)
CaPS	-
ELSA	1.01(0.61,1.69)
HAS	2.66(0.50,14.12)
HCS	1.13(0.51,2.50)
LBC1921	1.52(0.19,12.26)
NSHD	2.05(0.72,5.89)
WHII	1.39(0.69,2.82)
Combined Fixed Effect	1.14(0.82,1.58)
Combined Random Effect	1.14(0.82,1.58)
Estimated variance of random effect	5.50e-17(0.00e+00,.)

*p<0.05, **p<0.01, ***p<0.001, ****p<0.0001

[a] Coefficient is odds ratio for COPD in carriers versus non-carriers

Medium-chain acyl Co-A dehydrogenase deficiency

S41 Table. MCADD (K304E) carrier frequency

Cohort	Number of carriers[a]	Number of non-carriers[a]	Total, N[a]	Minor Allele Frequency	% female	Mean age[b] (SE)
BO	2	263	265	0.004	55.09	69.62(0.26)
CaPS	13	1237	1250	0.005	0	73.14(0.15)
ELSA	90	5393	5483	0.008	54.37	66.10(0.13)
HAS	8	511	519	0.008	38.92	76.39(0.16)
HCS	43	2729	2772	0.008	47.19	68.25(0.07)
LBC1921	0	522	522	0.000	58.24	79.07(0.03)
NSHD	33	2649	2682	0.006	50.15	53.45(0.00)
WHII	89	4379	4468	0.010	24.17	60.90(0.09)
Combined	278	17683	17961	0.008	41.01	64.83(0.09)

[a] Total number of individuals for each mutation is total number with a valid genotype, sex, age and at least one outcome measure
[b] Mean age at walk test for LBC1921 and WHII, and at balance test for all other cohorts

S25 Table. Association of K304E carrier status with physical capability, adjusted for age and sex

Outcome	Cohort	Coefficient[a] (95% CI)
Grip strength	ELSA	[c]
	HAS	
	HCS	
	LBC1921	
	NSHD	
	Combined Fixed Effect	-0.05(-0.15,0.04)
	Combined Random Effect	-0.05(-0.15,0.04)
	Estimated variance of random effect	4.60e-18[b]
Chair rise speed	ELSA	[c]
	HAS	
	HCS	
	NSHD	
	Combined Fixed Effect	0.10(-0.06,0.26)
	Combined Random Effect	0.10(-0.06,0.26)
	Estimated variance of random effect	1.05e-16(4.35e-37,2.53e+04)
Walk speed	ELSA	[c]
	HAS	
	HCS	
	LBC1921	
	WHII	
	Combined Fixed Effect	0.12(-0.02,0.26)
	Combined Random Effect	0.12(-0.02,0.26)
	Estimated variance of random effect	5.01e-17(0.00e+00,.)

TUG speed	BO	[c]
	CaPS	
	HAS	
	HCS	
	NSHD	
	Combined Fixed Effect	0.10(-0.13,0.33)
	Combined Random Effect	0.11(-0.13,0.34)
	Estimated variance of random effect	7.56e-20[b]
Inability to balance for 5s	BO	[c]
	CaPS	
	ELSA	
	HAS	
	HCS	
	NSHD	
	Combined Fixed Effect	0.92(0.55,1.56)
	Combined Random Effect	0.92(0.55,1.56)
	Estimated variance of random effect	2.85e-10(0.00e+00,.)

Continuous outcomes z-scored within cohorts
*p<0.05, **p<0.01, ***p<0.001, ****p<0.0001
[a] Coefficients are linear regression coefficients for continuous outcomes and odds ratios for binary outcomes
[b] Standard error of the variance of the random effect not estimable
[c] Cohort specific estimates are suppressed due to the small number of carriers

S26 Table. Association of K304E carrier status with cognitive capability, adjusted for age and sex

Outcome		Cohort	Regression Coefficient (95% CI)
Crystallized ability	Mill Hill	HAS	0.27(-0.44,0.98)
		WHII	-0.39***(-0.62,-0.17)
	Mill Hill[b]	WHII	-0.35**(-0.57,-0.12)
	NART	CaPS	0.03(-0.52,0.57)
		LBC1921	NC
		NSHD	0.15(-0.22,0.51)
		Combined Fixed Effect	F3CH
		Combined Random Effect	F3CH
		Estimated variance of random effect	F3CH
Fluid ability	AH4	CaPS	0.16(-0.38,0.70)
		HAS	-0.16(-0.85,0.54)
		WHII	-0.18(-0.40,0.04)
		Combined Fixed Effect	-0.13(-0.33,0.06)
		Combined Random Effect	-0.14(-0.33,0.06)
		Estimated variance of random effect	7.24e-14(0.00e+00,.)
	Semantic fluency	CaPS	-0.30(-0.83,0.24)
		ELSA	0.10(-0.10,0.29)
		NSHD	-0.21(-0.56,0.13)
		WHII	-0.10(-0.33,0.12)
		Combined Fixed Effect	-0.05(-0.18,0.09)
		Combined Random Effect	-0.05(-0.19,0.09)
		Estimated variance of random effect	1.66e-03(7.13e-10,3.85e+03)

	Phemic Fluency	LBC1921	NC
		WHII	-0.19(-0.42,0.04)
	Search Speed[c]	ELSA	0.01(-0.19,0.21)
		NSHD	0.15(-0.19,0.49)
	Word recall	ELSA	0.03(-0.15,0.22)
		NSHD	0.07(-0.28,0.42)
		WHII	-0.06(-0.29,0.16)
		Combined Fixed Effect	0.00(-0.13,0.14)
		Combined Random Effect	0.01(-0.13,0.14)
		Estimated variance of random effect	2.12e-20(1.12e-40,4.03e+00)
	FCRT[a]	CaPS	0.03(-0.52,0.58)
	Ravens Progressive Matrices	LBC1921	NC
	Logical Memory	LBC1921	NC

Outcomes z-scored within cohorts
F3CH: Fewer than 3 cohorts with adequate data to perform the meta-analysis
NC: No carriers
*p<0.05, **p<0.01, ***p<0.001, ****p<0.0001
[a] inverse transformed prior to z-scoring
[b] square transformed prior to z-scoring
[c] natural log transformed prior to z-scoring

Phenylketonuria

S27 Table. PKU mutation carrier status

Cohort	Number of carriers[a,c]	Number of non-carriers[a]	Total, N[a]	Minor Allele Frequency	% female	Mean age[b] (SE)
BO	1	263	264	0.002	54.92	69.64(0.26)
CaPS	7	1236	1243	0.003	0	73.11(0.15)
ELSA	30	5367	5397	0.003	54.2	66.06(0.13)
HAS	5	495	500	0.005	39.2	76.39(0.16)
HCS	13	2732	2745	0.002	47.25	68.26(0.07)
LBC1921	4	522	526	0.004	57.98	79.06(0.02)
NSHD	18	2622	2640	0.003	49.92	53.45(0.00)
WHII	33	4394	4427	0.004	24.01	60.88(0.09)
Combined	111	17631	17742	0.003	40.86	64.83(0.09)

[a] Total number of individuals is total number with a valid carrier status, sex, age and at least one outcome measure
[b] Mean age at walk test for LBC1921 and WHII, and at balance test for all other cohorts
[c] A carrier is defined as any individual who carries at least one minor allele of any of these three SNPs. A non-carrier is homozygous for the major allele at each of these three SNPs. If an individual had a missing genotype for one of the PKU SNPs, they were included in the analyses if they were a carrier but were excluded if they were a non-carrier based on the remaining PKU SNPs

S28 Table. Association of PKU mutation carrier status with physical capability, adjusted for age and sex

Outcome	Cohort	Coefficient[b] (95% CI)
Grip strength	ELSA	[a]
	HAS	
	HCS	
	LBC1921	
	NSHD	
	Combined Fixed Effect	-0.14(-0.29,0.01)
	Combined Random Effect	-0.14(-0.29,0.01)
	Estimated variance of random effect	1.80e-17(1.41e-68,2.30e+34)
Chair rise speed	ELSA	[a]
	HAS	
	HCS	
	NSHD	
	Combined Fixed Effect	-0.23(-0.49,0.02)
	Combined Random Effect	-0.33(-0.73,0.07)
	Estimated variance of random effect	6.88e-02(4.01e-04,1.18e+01)
Walk Speed	ELSA	[a]
	HAS	
	HCS	
	LBC1921	
	WHII	
	Combined Fixed Effect	-0.07(-0.29,0.16)
	Combined Random Effect	-0.07(-0.29,0.16)
	Estimated variance of random effect	1.01e-12(6.68e-28,1.54e+03)

TUG Speed	BO	[a]
	CaPS	
	HAS	
	HCS	
	NSHD	
	Combined Fixed Effect	-0.01(-0.36,0.34)
	Combined Random Effect	0.03(-0.39,0.45)
	Estimated variance of random effect	5.13e-02(5.35e-05,4.92e+01)
Inability to balance for 5s	BO	[a]
	CaPS	
	ELSA	
	HAS	
	HCS	
	NSHD	
	Combined Fixed Effect	0.76(0.31,1.87)
	Combined Random Effect	0.76(0.31,1.87)
	Estimated variance of random effect	1.32e-14(0.00e+00,.)

Continuous outcomes z-scored within cohorts
*p<0.05, **p<0.01, ***p<0.001, ****p<0.0001
[a] Cohort specific estimates are suppressed due to the small number of carriers
[b] Coefficients are linear regression coefficients for continuous outcomes and odds ratios for binary outcomes

S29 Table. Association of PKU mutation carrier status with cognitive capability, adjusted for age and sex

Outcome		Cohort	Regression Coefficient (95% CI)
Crystallized ability	Mill Hill	HAS	-0.17(-1.06,0.72)
		WHII	0.02(-0.35,0.40)
	Mill Hill [b]	WHII	0.01(-0.36,0.39)
	NART	CaPS	0.36(-0.38,1.10)
		LBC1921	0.29(-0.69,1.27)
		NSHD	0.09(-0.38,0.56)
		Combined Fixed Effect	0.18(-0.18,0.55)
		Combined Random Effect	0.18(-0.18,0.55)
		Estimated variance of random effect	3.50e-18(0.00e+00,.)
Fluid ability	AH4	CaPS	0.32(-0.41,1.06)
		HAS	0.09(-0.79,0.97)
		WHII	-0.12(-0.48,0.25)
		Combined Fixed Effect	-0.01(-0.32,0.30)
		Combined Random Effect	-0.02(-0.32,0.29)
		Estimated variance of random effect	3.83e-15(2.95e-38,4.97e+08)
	Semantic fluency	CaPS	0.10(-0.63,0.82)
		ELSA	-0.45**(-0.79,-0.11)
		NSHD	0.15(-0.31,0.61)
		WHII	-0.12(-0.49,0.25)
		Combined Fixed Effect	-0.17(-0.38,0.05)

		Combined Random Effect	-0.15(-0.41,0.10)
		Estimated variance of random effect	1.74e-02(1.35e-04,2.25e+00)
	Phonemic fluency	LBC1921	-0.34(-1.34,0.65)
		WHII	-0.08(-0.47,0.30)
	Search Speed [c]	ELSA	-0.02(-0.36,0.33)
		NSHD	-0.20(-0.66,0.26)
	Word recall	ELSA	0.04(-0.28,0.36)
		NSHD	0.09(-0.37,0.55)
		WHII	-0.07(-0.44,0.30)
		Combined Fixed Effect	0.01(-0.20,0.23)
		Combined Random Effect	0.01(-0.20,0.23)
		Estimated variance of random effect	2.03e-19(7.80e-44,5.28e+05)
	FCRT[a]	CaPS	0.25(-0.53,1.03)
	Ravens Progressive Matrices	LBC1921	0.52(-0.46,1.50)
	Logical Memory	LBC1921	-0.33(-1.32,0.66)

Outcomes z-scored within cohorts
*p<0.05, **p<0.01, ***p<0.001, ****p<0.0001
[a] inverse transformed prior to z-scoring
[b] square transformed prior to z-scoring
[c] natural log transformed prior to z-scoring

S30 Table. Tests for equality of variances

Outcome	Groups tested	p-value		
		Levene's test	Brown & Forsythe's test (median)	Brown & Forsythe's test (trimmed mean)
FVC	deltaF508 carrier vs non-carrier	0.5	0.49	0.49
FVC	All PI classes	0.06	0.05	0.07
	PI-MS vs PI-MM	0.57	0.55	0.55
	PI-MZ vs PI-MM	<0.005	<0.005	<0.005
	PI-SS vs PI-MM	0.89	0.88	0.9
	PI-SZ vs PI-MM	0.83	0.82	0.82
	PI-ZZ vs PI-MM	0.5	0.32	0.5
SQRT(FVC)	All PI classes	0.26	0.22	0.27
	PI-MS vs PI-MM	0.44	0.45	0.45
	PI-MZ vs PI-MM	0.02	0.02	0.02
	PI-SS vs PI-MM	0.99	1	0.98
	PI-SZ vs PI-MM	0.84	0.85	0.83
	PI-ZZ vs PI-MM	0.68	0.41	0.68
FEV1	All PI classes	0.31	0.28	0.3
	PI-MS vs PI-MM	0.44	0.45	0.44
	PI-MZ vs PI-MM	0.03	0.03	0.03
	PI-SS vs PI-MM	0.55	0.5	0.55
	PI-SZ vs PI-MM	0.52	0.37	0.47
	PI-ZZ vs PI-MM	0.7	0.93	0.7
Height	All PI classes	0.48	0.52	0.46
	PI-MS vs PI-MM	0.27	0.28	0.27

	PI-MZ vs PI-MM	0.78	0.78	0.79
	PI-SS vs PI-MM	0.42	0.51	0.38
	PI-SZ vs PI-MM	0.65	0.65	0.65
	PI-ZZ vs PI-MM	0.12	0.13	0.12
Lung function measures were included as z-scores, height was included in cm. Tests performed using Stata's(18) –robvar- command , pooling cohorts and restricting to individuals with sex and age variables.				

S31 Table. Detecting influential data points in associations of interest

Association of interest (fixed effects analysis)	Minimum dfbeta statistic	Maximum dfbeta statistic	Regression Coefficient (95% CI) of carrier effect with most extreme 60 values removed
Association of PI-MZ vs PI-MM with FEV1, age and sex adjusted	-0.16	0.11	0.16****(0.09,0.22)
Association of PI-MZ vs PI-MM with FVC, age and sex adjusted	-0.15	0.14	0.16****(0.10,0.22)
Association of PI-MZ vs PI-MM with FEV1, age, sex, height and height-squared adjusted	-0.18	0.12	0.09**(0.04,0.15)
Association of PI-MZ vs PI-MM with FVC, age, sex, height and height-squared adjusted	-0.15	0.19	0.08**(0.02,0.13)
Association of PI-MZ vs PI-MM with height, age and sex adjusted	-0.13	0.1	1.55****(1.06,2.03)
Association of PI-MZ vs PI-MM with height, age, sex, FEV1 and FVC adjusted	-0.13	0.15	0.92***(0.45,1.38)
Association of PI-MZ vs PI-MM with ln(weight), age and sex adjusted	-0.11	0.17	0.01(-0.00,0.02)
Association of deltaF508 carrier status with FVC, age, sex, height and height-squared adjusted	-0.22	0.18	-0.07*(-0.14,-0.01)
Association of PI-MZ vs PI-MM with grip strength, age and sex adjusted	-0.15	0.25	0.06*(0.00,0.12)

Lung function and grip strength included as z-scores.
Height in cm and weight in kg
*p<0.05, **p<0.01, ***p<0.001, ****p<0.0001

Supplemental References

1. Belsley DA, Kuh E, Welsch RE. Regression Diagnostics: Identifying Influential Data and Sources of Collinearity. New York: Wiley; 1980.
2. Martin RM, Gunnell D, Pemberton J, Frankel S, Davey Smith G. Cohort Profile: The Boyd Orr cohort—an historical cohort study based on the 65 year follow-up of the Carnegie Survey of Diet and Health (1937–39). International Journal of Epidemiology. 2005;34(4):742-9.
3. Steptoe A, Breeze E, Banks J, Nazroo J. Cohort Profile: The English Longitudinal Study of Ageing. International Journal of Epidemiology. 2013;42(6):1640-8.
4. Syddall HE, Simmonds SJ, Martin HJ, Watson C, Dennison EM, Cooper C, et al. Cohort profile: The Hertfordshire Ageing Study (HAS). International Journal of Epidemiology. 2010;39(1):36-43.
5. Syddall H, Aihie Sayer A, Dennison E, Martin H, Barker D, Cooper C, et al. Cohort Profile: The Hertfordshire Cohort Study. International Journal of Epidemiology. 2005;34(6):1234-42.
6. Deary IJ, Gow AJ, Pattie A, Starr JM. Cohort Profile: The Lothian Birth Cohorts of 1921 and 1936. International Journal of Epidemiology. 2012;41(6):1576-84.
7. Kuh D, Pierce M, Adams J, Deanfield J, Ekelund U, Friberg P, et al. Cohort Profile: Updating the cohort profile for the MRC National Survey of Health and Development: a new clinic-based data collection for ageing research. International Journal of Epidemiology. 2011;40(1):e1-e9.
8. Marmot M, Brunner E. Cohort Profile: The Whitehall II study. International Journal of Epidemiology. 2005;34(2):251-6.
9. Cooper R, Hardy R, Aihie Sayer A, Ben-Shlomo Y, Birnie K, Cooper C, et al. Age and Gender Differences in Physical Capability Levels from Mid-Life Onwards: The Harmonisation and Meta-Analysis of Data from Eight UK Cohort Studies. PLoS ONE. 2011;6(11):e27899.
10. Nelson HE, Willison JR. National Adult Reading Test (NART) test manual (Part II). Windsor, England: NFER-NELSON; 1991.
11. Raven JC. Guide to using the Mill Hill vocabulary test with progressive matrices. London, England: HK Lewis; 1965.
12. Heim AW. AH4 Group Test of General Intelligence. Windsor: The NFER-Nelson Publishing Company; 1970.
13. Wechsler D. Wechsler Memory Scale-Revised. New York, NY: Psychological Corp; 1987.
14. Raven JC, Court JH, Raven J. Manual for Raven's Progressive Matrices and Vocabulary Scales. London, England: H. K. Lewis; 1977.
15. Bolton CE, Cockcroft JR, Sabit R, Munnery M, McEniery CM, Wilkinson IB, et al. Lung function in mid-life compared with later life is a stronger predictor of arterial stiffness in men: The Caerphilly Prospective Study. International Journal of Epidemiology. 2009;38(3):867-76.
16. Wadsworth M, Kuh D, Richards M, Hardy R. Cohort Profile: The 1946 National Birth Cohort (MRC National Survey of Health and Development). International Journal of Epidemiology. 2006;35(1):49-54.
17. Wadsworth MEJ, Vinall LE, Jones AL, Hardy RJ, Whitehouse DB, Butterworth SL, et al. Alpha1-Antitrypsin as a Risk for Infant and Adult Respiratory Outcomes in a National Birth Cohort. American Journal of Respiratory Cell and Molecular Biology. 2004;31(5):559-64.
18. StataCorp. Stata Statistical Software: Release 13. College Station, TX: StataCorp LP; 2013.
